# The F1F3 recombinant chimera induced higher vaccine efficacy than its independent F1 and F3 components against *Leishmania* (*L.*) *infantum chagasi* mice infection

**DOI:** 10.3389/fimmu.2025.1598755

**Published:** 2025-07-01

**Authors:** Daniele Crespo Gomes, Maria Paula Fonseca-Ribeiro, Marcus Vinicius Alves-Silva, Clarisa B. Palatnik-de-Sousa

**Affiliations:** Laboratório de Biologia e Bioquímica de Leishmania, Instituto de Microbiologia Paulo de Góes, Departamento de Microbiologia Geral, Universidade Federal do Rio de Janeiro, Rio de Janeiro, Brazil

**Keywords:** *Leishmania* (*L.*) *infantum chagasi*, visceral leishmaniasis, nucleoside hydrolase NH36, F1F3 recombinant chimera, mixed or T-cell regulatory response

## Abstract

**Introduction:**

Visceral leishmaniasis (VL) is a severe human vector-borne CD4-immunosuppressive disease that can be lethal if untreated soon after symptoms arise. No vaccine is available against human VL, and its chemotherapy is highly toxic and requires hospitalization. VL patients show substantially decreased CD4_+_ total and *Leishmania*-specific CD4+ T cell counts. *Leishmania (L.) donovani* nucleoside hydrolase (NH36) is a DNA metabolism enzyme and a conserved marker of the *Leishmania* genus. It has been considered, among other *Leishmania* antigens, a vaccine candidate. In mice vaccinated with NH36, protection against VL is mediated by a CD4_+_ T cell response to the NH36 C-terminal domain (F3), and against cutaneous leishmaniasis (CL), by a CD4+ response against F3 and a CD8+ response against the NH36 N-terminal (F1). Vaccination with a recombinant chimera containing the F1 and F3 domains expressed in tandem (F1F3) protected mice against the heterologous CL infection by *L. (L.) amazonensis* and *L. (V.) braziliensis*.

**Methods:**

In this investigation, BALB/c mice were immunized with either F1, F3, a mixture of both, or with the F1F3 chimera, plus saponin and challenged with amastigotes of *L. (L.) infantum chagasi*, the agent of VL in America.

**Results:**

Before and after infection, the F1F3 chimera and the F3 vaccines promoted the highest IgA, IgM, IgG, IgG1, IgG2a, IgG2b, and IgG3 antibody responses. The F1F3 chimera promoted the strongest intradermal response against the leishmanial antigen, the highest body weight gain, and the most potent reduction of the spleen and liver relative weights. In addition, the F1F3 chimera vaccine increased the secretion of IFN-γ, and, together with the F3 vaccine, the secretion of TNF-α by splenocytes. The F1F3 chimera and the F1 vaccine also promoted the strongest secretion of IL-10, which was very low in mice immunized with F3. Thus, the IFN-γ/IL-10 and TNF-α/IL-10 ratios, characteristic of a Th1 response, were increased in mice vaccinated with F3. The F1F3 chimera and the F3 vaccine reduced the parasite load in the liver.

**Discussion:**

The F1F3 chimera, as described for the heterologous CL infections, also optimizes protection against the homologous visceral leishmaniasis infection by *L. (L.) infantum chagasi*, by a Th1 contribution from the F3 peptide and a regulatory response from the F1 peptide. Expression of the F1 and F3 domains *in tandem* induced higher efficacy than the simple mixture of the F1 and F3 domains.

## 1 Introduction

Visceral leishmaniasis (VL), the most severe form of leishmaniasis, is a human vector-borne protozoan disease caused by parasites of the *Leishmania* genus, which is almost always fatal if untreated soon after the rise of the symptoms. While 700,000 to 1,000,000 new leishmaniasis cases are registered annually, approximately 50,000 to 90,000 cases correspond to VL, although these might be underestimated. Most of them occur in Brazil, Eastern Africa, and the Indian subcontinent (1). VL is anthroponotic in Eastern Africa and the Indian Subcontinent (ISC), and it is a canid zoonosis in South America, the Mediterranean basin, China, and the Middle East (2, 3). High lethality and relapses are reported in Brazil and East Africa (4–6). Fever, weight loss, spleen and liver enlargement, anemia, hypergammaglobulinemia, and progressive suppression of the CD4^+^ total and CD4^+^ *Leishmania*-specific cellular immune response characterize human VL.

On the other hand, cutaneous leishmaniasis (CL) is the most frequent form of leishmaniasis that causes skin lesions, mainly ulcers, on exposed parts of the body. These can leave lifelong scars and cause severe disability or stigma. About 95% of the 600,000 new annual CL cases occur in the Americas, the Mediterranean basin, the Middle East, and Central Asia (1). Brazil has the highest incidence of CL in America and is one of the 10 countries that exhibit the highest number of CL cases in the world. Furthermore, mucocutaneous leishmaniasis (MCL) leads to partial or total destruction of the mucous membranes of the nose, mouth, and throat. Over 90% of mucocutaneous leishmaniasis cases occur in Bolivia, Brazil, Ethiopia, and Peru (1).

Since the areas affected by VL, CL, and MCL overlap significantly geographically and no preventive vaccine is available yet against any form of human leishmaniasis, it would be worthwhile to develop a cross-protective vaccine based on a *Leishmania*-conserved antigen.

*Leishmania* (*L*.) *donovani* nucleoside hydrolase NH36 is a vital enzyme of the parasite’s metabolism (7) and, consequently, an essential phylogenetic marker of the *Leishmania* genus (8, 9). *Leishmania* (*L*.) *donovani* is the agent of VL in India and Central Africa. The NH36 of *Leishmania* (*L*.) *donovani* shares high levels of sequence identity with nucleoside hydrolases (NHs) of *L.* (*L*.) *infantum chagasi* (99%), the agent of VL of the Americas, with NHs of *L.* (*L*.) *amazonensis* (92%), *L.* (*V*.) *braziliensis* (84%), *L.* (*L*.) *mexicana* (93%), and *L.* (*V*.) *guyanensis* (84%), the agents of cutaneous leishmaniasis of America (ACL), and with NHs of *L.* (*L*.) *tropica* (97%) and *L.* (*L*.) *major* (95%–96%), the agents of CL of the Old World (10–14). Therefore, NH36 became an excellent candidate for conserved antigens for powerful vaccines that could exert bivalent protection against VL and CL of humans and animals.

Previous studies (15) demonstrated that the F3 domain of NH36 formulated with saponin protects mice against VL caused by *Leishmania* (*L*.) *infantum chagasi*, with a response mediated by CD4^+^ T cells and a small contribution from CD8^+^ T cells. The F3 domain was the most potent immune-protective fraction of NH36 and reduced the parasite load by 88% (15). In contrast, in the vaccination of mice against cutaneous leishmaniasis caused by *Leishmania* (*L*.) *amazonensis*, the F1 domain was the one that showed the most substantial reduction in lesion size with a response mediated by CD8^+^ T cells (16).

Recent approaches support that vaccination with a combination of the most immunogenic fractions of a protein can optimize vaccine efficacy (17–19)—for instance, the only current vaccine licensed for the prevention of canine VL in Europe, called LetiFend^®^, is composed of a recombinant chimeric protein (Protein Q) formed by the genetic fusion of five antigenic fragments from the Lip2a, Lip2b, H2A, and P0 proteins (20) and has shown efficacy and safety in a recent canine clinical assay (21).

Furthermore, the chimera vaccines, being more protective than their isolated component fractions, could also determine cross-immunity against diverse variants, strains, or species of pathogens that cause the disease in different geographical regions, conferring, in that way, universal protection. With this objective in mind, we previously studied the immunoprotective potential of the F1 and F3 domains of NH36 expressed in tandem in the recombinant F1F3 chimera in the prevention of the heterologous cutaneous infections caused in mice by *L.* (*L*.) *amazonensis* (10) or *L.* (*V*.) *braziliensis* (22). When compared with the F1 or F3 independent domains, vaccination with the F1F3 chimera promoted a more substantial reduction of *L.* (*L*.) *amazonensis* (10) and *L.* (*V*.) *braziliensis* (22) parasite loads (99.9% and 99.8%, respectively) and lesion sizes (84% and 62%, respectively).

Aiming to extend the study of the cross-protective capabilities of the F1F3 chimera, in this investigation, we compared its immunoprotective potential to that of the F1 and F3 domains individually or combined in a simple mixture in the prevention of the homologous visceral infection of mice by the American agent of VL, *Leishmania* (*L*.) *infantum chagasi*.

## 2 Materials and methods

### 2.1 Ethical statement

This study was approved by the Ethics Committee on the Use of Animals of the Federal University of Rio de Janeiro (CEUA-UFRJ) (IMPPG no. 44). To minimize animal suffering, all of the experiments were performed according to the recommendations of the National Institutes of Health (NIH) and the Brazilian laws for animal safety. The animals were fed *ad libitum* and maintained under controlled temperature with 12-h dark/light cycles at the Instituto de Microbiologia Paulo de Góes, Universidade Federal do Rio de Janeiro (UFRJ) facilities. The animals were euthanized following the guidelines established by the National Council for the Control of Animal Experimentation (CONCEA). The procedure followed the protocol recommended by CONCEA, with a combination of dissociative anesthetics ketamine (250 mg/kg) and xylazine (50 mg/kg) being administered intraperitoneally at a dose three times higher than that used for anesthetic induction to promote humane euthanasia. Death was confirmed in a specific chamber with a controlled atmosphere of carbon dioxide (CO_2_) using a gradual flow equivalent to 20% of the chamber volume per minute, with 100% CO_2_. The flow was maintained for at least 1 min after verifying the absence of clinical signs of life, such as respiratory movements and heartbeats.

### 2.2 Recombinant antigen expression and purification

The sequence of the nucleoside hydrolase (NH36) (314 amino acids) has Genbank AY007193 and SwissProt-UniProt Q8WQX2-LEIDO accession numbers. The F1 domain represents the N-terminal domain of NH36, which is composed of its first 103 amino acids. The F3 domain represents the NH36 C-terminal moiety and is composed of amino acids 199 to 314 in the sequence (15). The F1F3 chimera comprises the F1 and F3 domains linked in tandem. All of the antigens were cloned in the pET28b expression vector between the sites of *Nco*I and *Xho*I and were terminated by a sequence of six histidine residues at their C-termini. For the expression of the recombinant antigens, *Escherichia coli* BI21 (DE3) bacteria cells were transformed with the plasmids pET28bF1, pET28bF3, or pET28bF1F3 (10, 22). Briefly, we placed 1 mL of each culture in 12 mL of LB medium supplemented with 30 µg/mL kanamycin and incubated them in a shaker at 250 × *g* and 37°C. When the suspensions reached an OD of 0.6–0.8 at 600 nm, 1 mM of IPTG (isopropyl-beta-D-thiogalactopyranoside) was added to induce protein expression, and the suspensions were incubated for an additional 4 h. After that, the cultures were further centrifuged for 20 min at 5,000 × *g* and 4°C, the supernatants were discarded, and the bacterial pellets were stored at -20°C. For purification, the bacterial pellets were mixed with 20 mL of sonication buffer (7.52 g NaCl, 13.8 g NaH2PO4, 1 mM PMSF, and 10 mg lysozyme), kept on an ice bath, and sonicated for 20 cycles of 5 s, with an interval of 10 s, in a Fisher Scientific Sonic Dismembrator model 500. After that, the sonicated material was centrifuged for 20 min at 15,000 × *g* and 4°C. The supernatants were discarded since the F1, F3, and F1F3 antigens remained more concentrated in the insoluble fraction of the bacterial pellets. Furthermore, the antigens were purified according to the instructions of the Ni-NTA resin manufacturer (Qiagen) (10, 22). The proteins were finally dialyzed against 50 mM Tris-HCl, pH 8, 50 mM NaCl, 50% glycerol, and 0.1 mM DTT, and the absence of LPS was confirmed using the LAL QCL-1000 kit (Lonza). SDS PAGE confirmed the purity of the recombinant proteins on 15% polyacrylamide gels stained with Coomassie Brilliant Blue-R-250 (Bio-Rad, USA).

### 2.3 Mice vaccination, challenge, and clinical and parasitological outcomes

Female BALB/c mice at 2 to 4 months old were randomized according to their weight (range: 15 to 35.5 g) and vaccinated subcutaneously on the back, at weekly intervals, with three injections of 100 µg of F1, F3, the F1 + F3 mixture, or 100 or 200 µg of the recombinant F1F3 chimera. The sample size for each treatment was *n* = 26, corresponding to two identical experiments, each with 13 mice per treatment. The mix of F1 + F3 contained 50 µg of F1 and 50 µg of F3. Each vaccine was formulated with 100 µg of Riedel De Haen Saponin (Sigma) in 0.2 mL of NaCl 0.9% saline solution. The control mice received only saline. At 1 week after the complete immunization schedule, sera samples were collected, and an intradermal reaction (IDR) against *L.* (*L*.) *donovani* (LD-1S/MHOM/SD/00-strain 1S) lysate was performed. After the IDR, all animals were intravenously inoculated with 3 × 10–^7^ *Leishmania* (*L*.) *infantum chagasi* (strain IOC-L 3324) infective amastigotes obtained from hamster’s spleens.

At 15 days after infection, sera samples were collected again, another intradermal test was performed, and 48 h after that, all animals were euthanized. Corporal, spleen, and liver weights were assessed, the parasite load was evaluated, and mice splenocytes were incubated with NH36 to evaluate the expression of cytokines in the supernatants. The increments of corporal weight, the spleen/body relative weight, the liver/body relative weight, and the liver parasite burden were considered major clinical outcomes to calculate the vaccine’s efficacy. The parasite load was determined by optical microscope observation of liver smears stained with Giemsa dye using a ×100 immersion objective and expressed as Leishman Donovan units (LDU units) of Stauber = number of amastigotes/1,000 organ cell nuclei × organ weight in milligrams (15, 23, 24).

### 2.4 Antibody assays in sera

At 7 days after vaccination and 15 days after infection, blood was collected by tail bleeding, and sera were obtained. Plates were sensitized with 2 µg of recombinant NH36 protein in sodium bicarbonate buffer, pH 9.6, and incubated for 1 h at 37°C and overnight at 4°C. The plates were then washed with PBS** (PBS 0.018, pH 7.2, 1% milk, and 0.05% Tween 20), incubated with 1/100 serum diluted samples in PBS** for 1 h at 37°C, washed again with PBS**, and incubated with 50 μL of peroxidase-conjugated goat anti-mouse IgA, IgM, IgG, IgG1, IgG2a, IgG2b, or IgG3 antibodies (Southern Biotechnology Associates, Birmingham, AL, USA) or with 50 µL of peroxidase-protein-A pool (Kirkegaard & Perry Laboratories, Gaithersburg, MD, USA) at 1:1,000 dilution in PBS**. The plates were incubated for 1 h and washed five times with PBS**. The reactions were developed using an OPD buffer (ortho-phenylenediamine—Sigma), stopped with 1 N sulfuric acid, and recorded using a BioRad ELISA Reader at 492 nm filter. Each serum sample was analyzed in triplicate (25).

### 2.5 Delayed type of hypersensitivity

The intradermal response (IDR) to *Leishmania* (*L*.) *donovani* stationary-phase promastigote lysate was evaluated after sera collection on day 7 after vaccination and day 15 after infection. The promastigotes were cultured *in vitro* in 200 mL of Schneider’s (Sigma) medium supplemented with 10% fetal calf serum (Cultilab, SP, Brazil) under 150 × *g* at 28°C for 3 days until they reached the stationary growth phase. The parasites were centrifuged, washed with saline solution, counted in Neubauer chambers to prepare a suspension of 10^8^ promastigotes/mL, frozen in liquid nitrogen, and thawed under a stream of running water five times so that cell lysis could take place.

To evaluate the IDR response, the animals were inoculated with 100 µL of the lysate (10^7^ *L.* (*L*.) *donovani* promastigotes) in the footpad of the right hind paws. We used 100 µL of saline solution as a negative control in the left hind paws. Paw measurements (five measurements per animal) were conducted using a Mitutoyo caliper at times 0, 24, and 48 h after the inoculation of the lysate. The IDR response was measured as the difference in paw thickness before and after lysate inoculation at each time point. For each mean, the values of its respective negative control paws, injected with saline, were subtracted (25).

### 2.6 Cytokine assays

Spleens were aseptically removed after euthanasia, and splenocytes were obtained through maceration with the plunger of a syringe. The macerate was placed in polystyrene tubes containing ACK solution (0.15 M ammonium chloride, 0.01 M potassium bicarbonate, and 0.0001 M EDTA) and centrifuged at 250 × *g* for 5 min repeatedly until all red blood cells were removed. Then, the single-cell suspensions were washed with saline solution, suspended in 1 mL of RPMI medium (Sigma, Co) supplemented with 10% fetal bovine serum (Nutricell, Campinas SP), 1% L-glutamine, and 5 mM β-mercaptoethanol, and counted in a Neubauer chamber (10). The splenocytes were distributed and cultured on 96-well Costar plates at a concentration of 10^6^ splenocytes/well and exposed to 5 μg of recombinant NH36 per well for 5 days at 37°C. The cytokine assay followed the manufacturer’s instructions using the BD OptEIA Mouse TNF ELISA Set II, IFN-γ, and IL-10 ELISA Set II kits from BD Biosciences. The absorbance values were recorded in a Perkin-Elmer spectrophotometer with a 655-nm filter. The IFN-γ/IL-10 and TNF-α/IL-0 ratios were also calculated.

### 2.7 Statistical analysis

Means of antibody absorbance values were compared by using ANOVA and Dunn’s multiple comparisons. Means of IDR, secreted cytokines, weight gain, liver and spleen relative weights, and LDU values were compared using 95% confidence interval. A Pearson two-tailed bivariate test was conducted for correlation analysis using GraphPad Prism 6 software.

## 3 Results

### 3.1 Recombinant antigens

The recombinant proteins were solubilized with urea and purified on a NiNTA column. Protein concentration was assayed using the Lowry method and analyzed by electrophoresis in 15% polyacrylamide gel (**Supplementary Figure S1**). All proteins were successfully expressed. From left to right, we show the molecular weight standards, the NH36 antigen (estimated molecular weight = 34.2387 kDa), the F1 and F3 domains, the F1F3lab chimera cloned in our lab with non-optimized codons, and the F1F3GS chimera, cloned by Genscript with optimized codons for *E. coli*. The optimized chimera F1F3GS shows a molecular weight (m.w.) of approximately 30 kDa, while the non-optimized chimera has a slightly higher m.w. It is worth noting that the expression of the F1F3lab chimera was feeble. In effect, while the yield of each batch of the optimized F1F3 chimera, cloned by Genscript, was 8 mg/L with just 4 h of induction, an induction time of 24 h was necessary to obtain 0.333 mg/L of the F1F3Lab chimera. For this reason, the mice were vaccinated using the F1F3GS chimera. F1 has 103 amino acids and an estimated molecular weight = 10.8456 kDa. F3 contains 116 amino acids and has an estimated molecular weight = 13.1012 kDa. The F1F3 chimera is therefore composed of the F1 domain in its N-terminal and the F3 domain in its C-terminal, contains 219 amino acids, and has an estimated molecular weight of 23.9287 kDa. Furthermore, the absence of LPS endotoxin was confirmed using the LAL QCL-1000 kit (Lonza). The detected concentrations of LPS were below 0.1 EU/mL and therefore considered below the assay’s detection limit, effectively indicating the absence of detectable endotoxin.

### 3.2 The F1F3 chimera and the F3 vaccines optimize the antibody response

**Figure 1:**
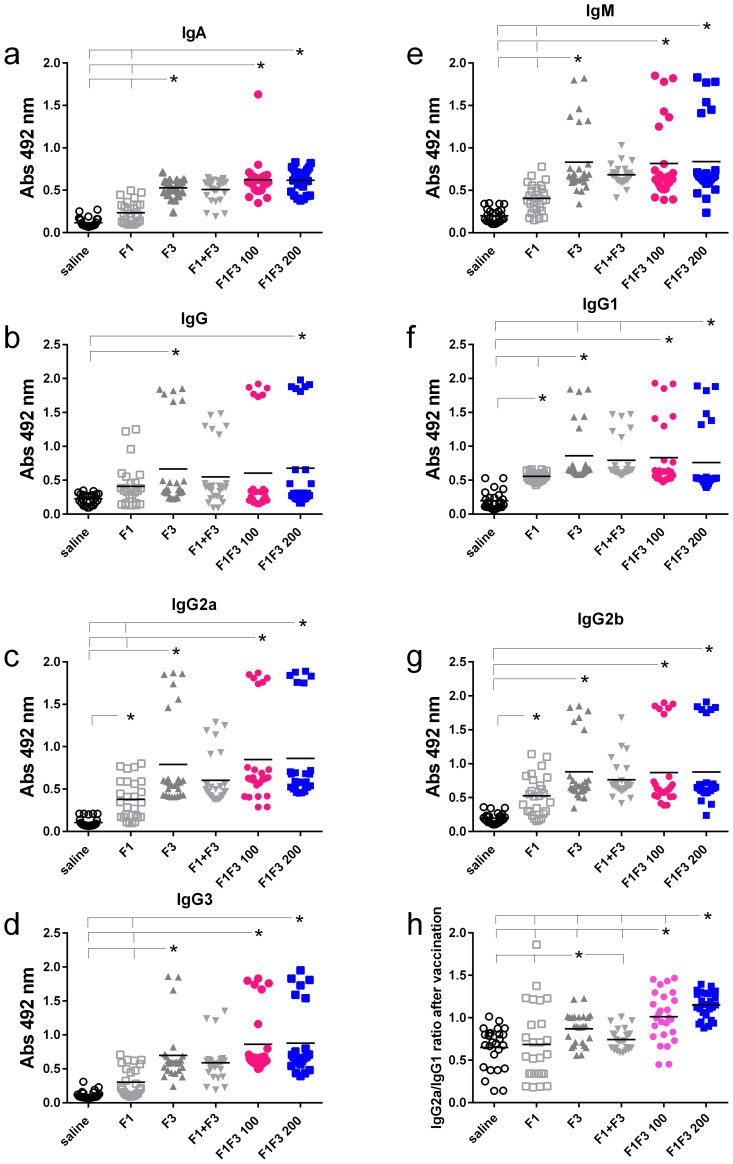
Predominance of NH36-antibody responses promoted by the F3 and chimeras after vaccination. The mice were vaccinated with the F1, F3, F1+F3 mixture, or F1F3 chimeras at 100 µg or 200 µg doses, all formulated with 100 µg saponin. Results represent the individual absorbance data of anti-IgA **(a)**, IgG **(b)**, IgG2a **(c)**, IgG3 **(d)**, IgM **(e)**, IgG1 **(f)** and IgG2b **(g)** anti-NH36 antibodies in mice sera diluted 1/100, as measured by the ELISA assay, and the individual IgG2a/IgG1 antibody ratios **(h)**. Statistical differences were evaluated using the Kruskal-Wallis method and ANOVA. Horizontal bars represent the means of two independent experiments with n = 26 mice per treatment. Asterisks and horizontal lines indicate significant differences between treatments (p < 0.001).

**Figure 2:**
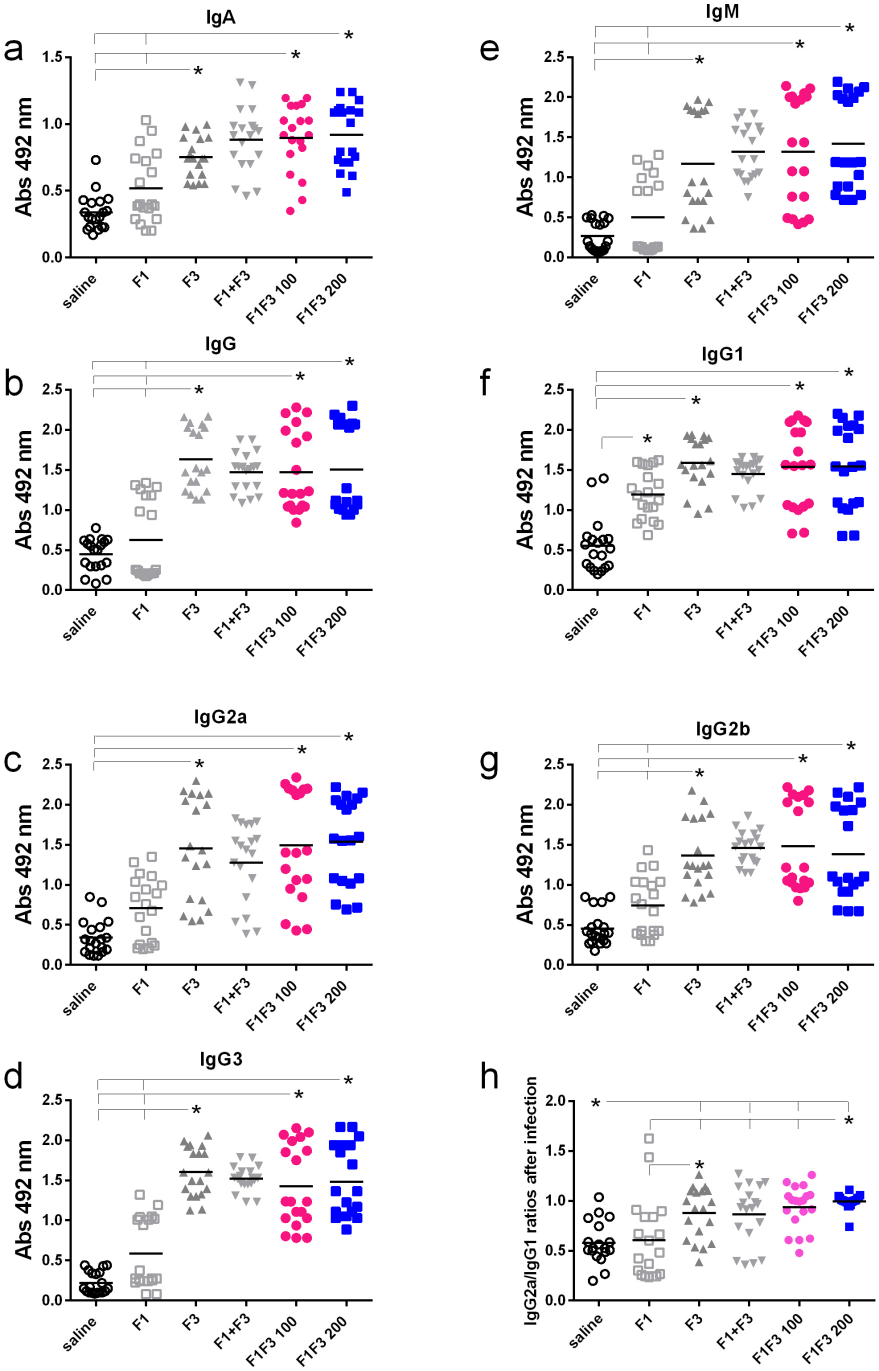
Predominance of NH36-antibody responses promoted by the F3 and chimeras after infection. Mice were vaccinated with the F1, F3, F1 + F3 mixture, or F1F3 chimeras at 100- or 200-µg doses, all formulated with 100 µg saponin. The results represent the individual absorbance data of anti-IgA **(a)**, IgG **(b)**, IgG2a **(c)**, IgG3 **(d)**, IgM **(e)**, IgG1 **(f)**, and IgG2b **(g)** anti-NH36 antibodies in mice sera diluted 1/100, as measured by the ELISA assay, and the individual IgG2a/IgG1 antibody ratios **(h)**. Statistical differences were evaluated using the Kruskal–Wallis method and ANOVA. Horizontal bars represent the means of two independent experiments with *n* = 19 mice per treatment. Asterisks and horizontal lines indicate significant differences between treatments (*p* < 0.001).

We compared the anti-NH36 antibody response promoted by F1, F3, F1 + F3, and the F1F3 chimera vaccines. Both before (**Figures 1a–g**) and after infection (**Figures 2a–g**), the two concentrations of the F1F3 chimera (100 and 200 µg) and the F3 vaccines promoted the highest IgA, IgM, IgG, IgG1, IgG2a, IgG2b, and IgG3 antibody absorbance values. However, the F3 vaccine was slightly stronger than the F1F3–200 chimera only for IgG1 after vaccination (**Figure 1f**). In contrast, the F1 vaccine did not generate a major antibody response (**Figures 1** and **2**). The F1 vaccine absorbance values only differed from the saline controls in the IgG1, IgG2a, and IgG2b subtypes after immunization (**Figures 1f, c, g**) and in the IgG1 subtype after infection (**Figure 2f**). Our results also disclose that the association of F1 and F3 in tandem, in the chimera, raised a better antigen presentation to APCs than the simple mixture of the F1 and F3 domains (F1 + F3) (**Figures 1a–g**, **2a–g**). Furthermore, there was no significant difference between the two concentrations of the chimera, suggesting that 100 µg of the vaccine is sufficient to optimize the antibody vaccine-generated response (**Figures 1a–g**, **2a–g**). After vaccination, 200 µg of the chimera promoted higher IgG2a/IgG1 ratios than 100 µg, and both were more potent than the F3 vaccine (**Figure 1h**). After infection (**Figure 2h**), the F1F3 chimera (200 µg) induced the highest IgG2a/IgG1 ratios. F3 was more potent than the F1 vaccine, which was not different from the saline controls. These IgG2a/IgG1 antibody ratios result in the F1F3 chimera stimulation of a more substantial Th1 response than the F3 vaccine. In contrast, the F1 vaccine did not promote such a response. In addition, all of the vaccines, with the exception of F1 for IgM, IgG, IgG2a, IgG2b, and IgG3, promoted significant increases of antibody absorbance values after infection (*p* < 0.012). The F3 and the two dosages of the F1F3 vaccine, which were the most potent, exhibited absorbance increases after infection, with a range of 42%–49% for IgA, 41%–69% for IgM, 145%–122% for IgG, 84%–103% for IgG1, 84%–78% for IgG2a, 55%–58% for IgG2b, and 131%–69% for IgG3. These increases were, therefore, more pronounced for the IgG class, followed by the IgG1, IgG2a, and IgG2b subclasses (**Supplementary Figures S2a, b**). While F3 was more potent in IgG, IgG2a, and IgG3, one of the two chimeras predominated in IgA, IgM, IgG1, and IgG2b antibodies (**Supplementary Figures S2a, b**).

### 3.3 The F1F3 chimera promotes the highest IDR response

**Figure 3:**
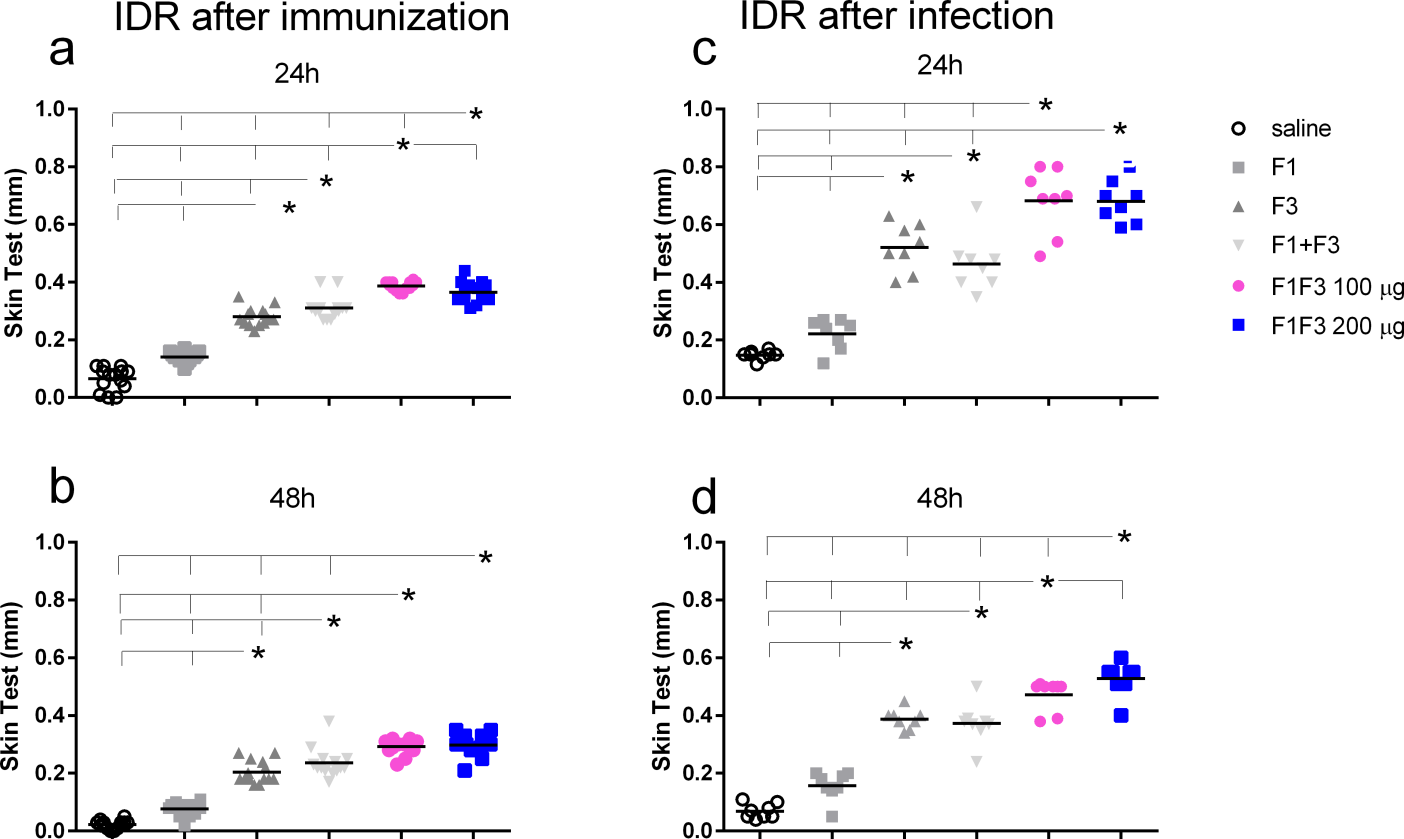
The F1F3 vaccine induces the strongest intradermal response. The mice were vaccinated with the F1, F3, F1 + F3 mixture, or F1F3 chimeras at 100- or 200-µg doses, all formulated with 100 µg saponin. The results represent the individual intradermal size response to the leishmanial antigen in millimeter after vaccination **(a, b)** and after infection **(c, d)**. Horizontal bars represent the means of two independent experiments with *n* = 14 mice per treatment after vaccination and *n* = 8 mice per treatment after infection. Asterisks and horizontal lines indicate significant differences between treatments as assessed using 95% confidence interval.

**Figure 4:**
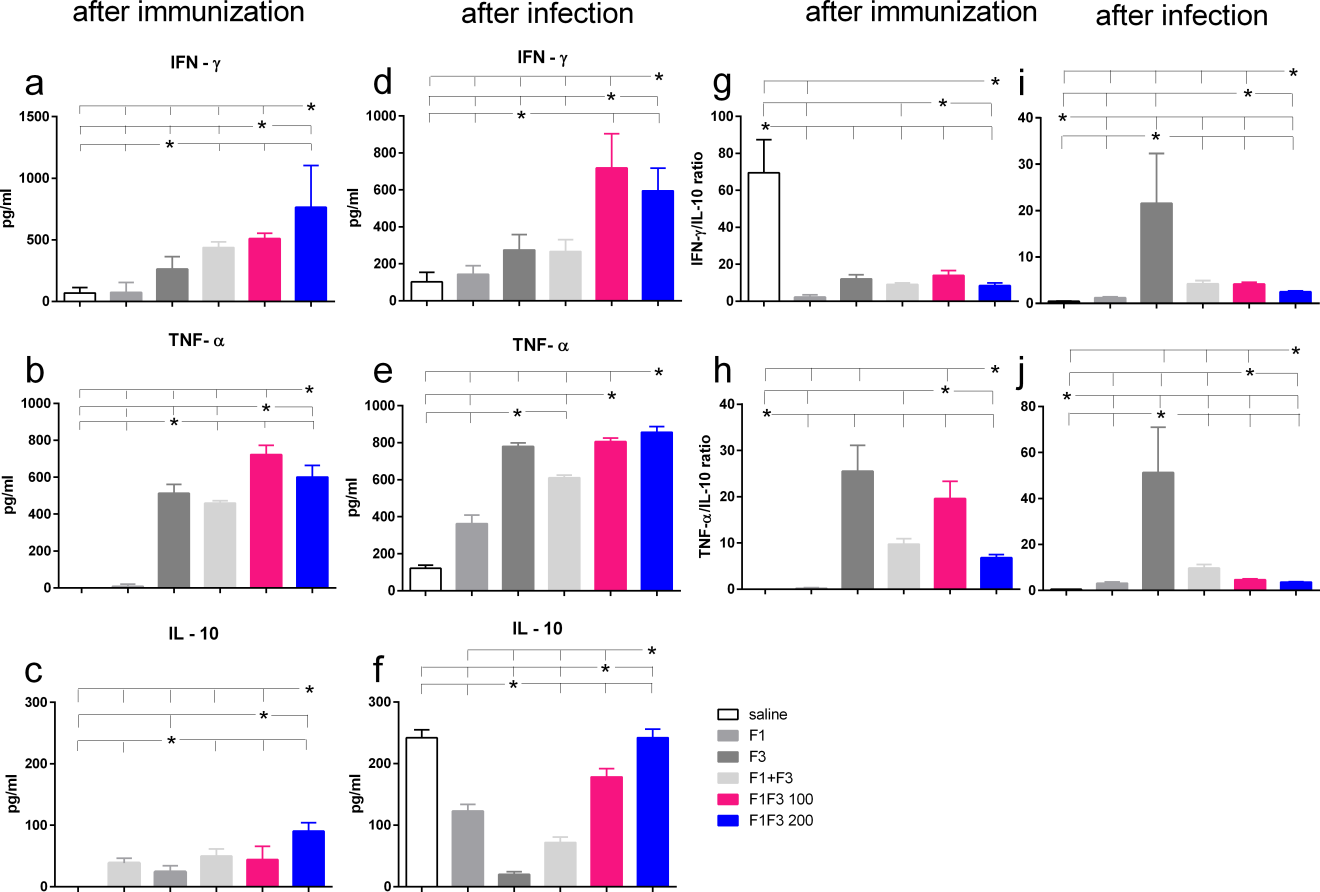
Th1-cytokine secretion promoted by the F3 vaccine and mixed inflammatory/regulatory cytokine response induced by the chimeras. The mice were vaccinated with the F1, F3, F1 + F3 mixture, or F1F3 chimeras at 100- or 200-µg doses, all formulated with 100 µg saponin. The results represent the means of IFN-γ **(a, d)**, TNF-α **(b, e)**, and IL-10 secretion **(c, f)** in response to NH36 after immunization and infection and the means of IFN-γ/IL10 **(g, i)** and TNF-α/IL10 ratios **(h, j)** secreted by splenocytes after immunization and after infection, as evaluated in the ELISA assay (expressed in pg/mL). Horizontal bars represent the means ± SE of two independent experiments with *n* = 6 mice per treatment after vaccination and *n* = 8 mice per treatment after infection. Asterisks and horizontal lines indicate differences between treatments as disclosed using 95% confidence interval.

After immunization, both concentrations of F1F3 chimera were more substantial than all the other vaccines (**Figures 3a, b**). A dose of 100 µg of the chimera showed the best performance at 24 h after antigen injection (**Figure 3a**), and at 48 h, both concentrations of chimera were equally effective (**Figure 4b**). It is noteworthy that the infection enhanced the IDR values induced by the vaccines, mainly at 24 h after injection (1.86-, 1.76- and 1.86-fold increases for F3, F1F3 100, and F1F3 200, respectively), although both chimeras still promoted the strongest responses. At 48 h, the F1F3–200 chimera vaccine was predominant (**Figure 3d**). In contrast, despite being composed of the exact domains, the F1 + F3 mixture was less potent than the chimeras and was only stronger than the F3 vaccine after immunization (**Figures 3a, b**). Furthermore, increases in IDR responses after infection were more pronounced in mice vaccinated with the F3 (108%), followed by the chimeras (89% and 100% for the 100 and the 200 µg/dose, respectively) and the F1 + F3 mixture (60%).

### 3.4 Th1-cytokines induced by the F3 and a mixed cytokine response promoted by the chimeras

After vaccination and after challenge, both chimeras promoted the global strongest cytokine secretion. F1F3–200 induced the highest secretion of IFN-γ and IL-10 (**Figures 4a, c, g, f**) and F1F3 100 the strongest TNF-α response (**Figures 4b, e**). After challenge, the chimeras induced an increase of IFN-γ levels (**Figure 4d**), and all vaccines amplified the TNF-α secretion (**Figure 4e**). It is worth noting that the F3 and the F1F3–100 chimera intensified the TNF-α secretion to a similar extent (**Figure 4e**). In contrast, the unvaccinated infected mice showed a high secretion of IL-10, which was also present in mice that received all of the vaccines containing the F1 domain. The F3 vaccine, in contrast, absolutely prevented the increase of IL-10 after immunization (**Figure 4c**) and after infection (**Figure 4f**), indicating that it induces a primary Th1 response. In agreement, after infection, the F3 vaccine determined the maximal IFN-γ/IL10 ratios (five- to eight-fold higher than the chimeras) (**Figure 4i**) and the most potent TNF-α/IL-10 ratios (eight- to 11-fold higher than the chimeras) (**Figure 4j**). Conversely, after infection, the F1F3 chimera vaccine at both doses promoted mixed cytokine secreting with a strong secretion of the pro-inflammatory IFN-γ and TNF-α (**Figures 4d, e**) and the regulatory IL-10 cytokines (**Figure 4f**).

### 3.5 Optimization of vaccine efficacy by the F1F3 chimeras

**Figure 5:**
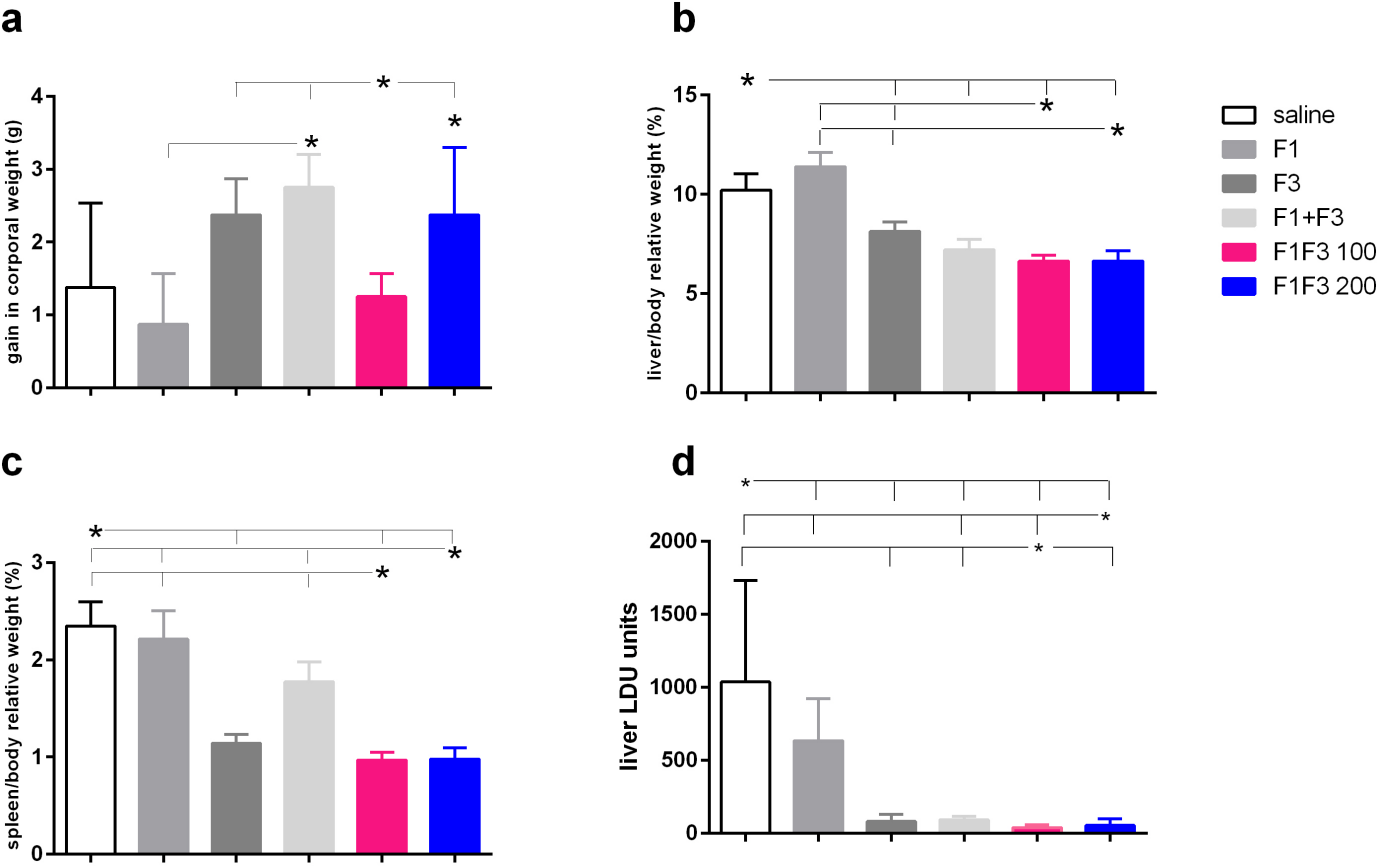
Impact of vaccination on clinical variables. Mice were vaccinated with the F1, F3, F1 + F3 mixture, or F1F3 chimeras at 100- or 200-µg doses, all formulated with 100 µg saponin. The results represent the gain in corporal weight in grams (g) **(a)**, the liver/body relative weight as percentages (%) **(b)**, the spleen/body relative weight in percentages (%), **(c)** and the parasite load in the liver of mice infected with amastigotes of *Leishmania* (*L*.) *infantum chagasi* expressed as LDU values **(d)** after euthanasia. Horizontal bars represent means ± SE of two independent experiments with *n* = 8–10 mice per treatment. Asterisks and horizontal lines indicate differences between treatments as assessed using 95% confidence interval.

The impact of vaccination on the clinical variables was studied at the time of euthanasia. The F1 + F3 mixture promoted more corporal weight gain than the F1 vaccine and, together with the F1F3–200 chimera and F3, determined more gain than the F1F3–100 vaccine (**Figure 5a**). There was, however, no difference between the vaccines and the saline control. Additionally, compared with the saline controls, both doses of the chimera promoted the most substantial reduction of liver relative weights. The F1F3–100 reduced by 35%, the F1F3–200 by 37.4% (**Figure 5b**), the F1 + F3 mixture by 29.4%, and the F3 vaccine by 20.2% of the liver relative weight while, in contrast, the F1 vaccine increased this variable by 11.6% (**Figure 5b**). Furthermore, the F1F3–100 and F1F3–200 chimeras reduced the relative weights of the spleens by 58.7% and 58.3%, respectively, and the F3 by 51.5% if compared to the saline controls (**Figure 5c**). In contrast, the F1 vaccine and the mixture F1 + F3 were not as efficient and showed reductions of 5.7% and 24.5%, respectively (**Figure 5c**). Accordingly, the evaluation of the parasite load in livers demonstrated that the F1F3–100 and F1F3–200 chimeras promoted reductions of 97.0% and 97.3%, respectively, the F3 95.1%, and the F1 + F3 mixture 94.2% when compared to the saline control (**Figure 5d**). Although the F1 vaccine was not effective in increasing corporal weight or reducing the spleen and liver relative weights, it reduced the parasite load by 59.0%, indicating that the evaluation of parasite burden is a more sensitive approach. Our results disclosed that we obtained vaccine efficacy optimization by combining the epitopes of the F1 and F3 domains. Both chimeras determined the strongest efficacy, protecting more than the mixture F1 + F3 in spleen relative weight and reduction of parasite load, indicating the benefit of presentation of both domains in tandem rather than in a simple mixture. In addition and supporting the achievement of optimization, the protection promoted by chimeras was higher than that induced by F3 in liver relative weight and in the reduction of parasite load (**Figure 5d**). Representative images of live smears of controls treated with saline and of F1F3 vaccinated mice are shown in **Supplementary Figures S3a, b**, respectively.

### 3.6 Correlation between immunological variables and clinical outputs

F1F3 vaccination effectively prevents *L.* (*L*.) *infantum chagasi* infection VL, as shown by strong correlations between immunological response and clinical evidence. As strong surrogates of protection, the IgM, IgG, IgG1, IgG2a, IgG2b, and IgG3 antibody absorbance values after infection were negatively and significantly correlated with the relative weights of the spleens (**Supplementary Figures S4a–f**) and livers (**Supplementary Figures S4g–1**) (**Table 1**). The absorbance values of all of these antibodies after vaccination, in addition to IgA, were also negatively correlated with the liver parasite load (**Table 2**) (**Supplementary Figures S5a–g**).

**Table 1:** Correlations between antibodies, intradermal DTH response, cytokines, and clinical outcomes.

Immunological variable	Clinical outcome	*p*	*R*	*R* ^2^
IgM after infection	Spleen relative weight	<0.0001	-0.6136	0.3765
IgG after infection	Spleen relative weight	<0.0001	-0.6151	0.3783
IgG1 after infection	Spleen relative weight	<0.0001	-0.6863	0.4710
IgG2a after infection	Spleen relative weight	0.0001	-0.5944	0.3533
IgG2b after infection	Spleen relative weight	0.0004	-0.5583	0.3117
IgG3 after infection	Spleen relative weight	<0.0001	-0.6181	0.3820
IgM after infection	Liver relative weight	0.0012	-0.5191	0.2694
IgG after infection	Liver relative weight	0.0015	-0.5100	0.2601
IgG1 after infection	Liver relative weight	0.0002	-0.5768	0.3327
IgG2a after infection	Liver relative weight	0.0026	-0.4862	0.2364
IgG2b after infection	Liver relative weight	0.0095	-0.4264	0.1818
IgG3 after infection	Liver relative weight	0.0015	-0.5109	0.2610
IDR 24 h after vaccination	Spleen relative weight	<0.0001	-0.6543	0.4282
IDR 48 h after vaccination	Spleen relative weight	<0.0001	-0.6567	0.4312
IDR 24 h after vaccination	Liver relative weight	0.0001	-0.5908	0.3490
IDR 48 h after vaccination	Liver relative weight	<0.0001	-0.6361	0.4046
IFN-γ after infection	Spleen relative weight	<0.0001	-0.6054	0.3665
TNF-α after infection	Spleen relative weight	<0.0001	-0.6896	0.4756
IFN-γ after infection	Liver relative weight	0.0012	-0.5188	0.2691
TNF-α after infection	Liver relative weight	0.0001	-0.5925	0.3511

**Table 2:** Correlations between antibodies, intradermal DTH response, cytokines, spleen, and liver relative weights and liver parasite loads.

Variable correlations	Clinical outcome	*p*	*R*	*R* ^2^
IgA after vaccination	Liver parasite load	0.0018	-0.5033	0.2533
IgM after vaccination	Liver parasite load	<0.0001	-0.6753	0.4561
IgG after vaccination	Liver parasite load	<0.0001	-0.7906	0.6250
IgG1 after vaccination	Liver parasite load	<0.0001	-0.7421	0.5507
IgG2a after vaccination	Liver parasite load	<0.0001	-0.7494	0.5616
IgG2b after vaccination	Liver parasite load	<0.0001	-0.7769	0.6036
IgG3 after vaccination	Liver parasite load	<0.0001	-0.6533	0.4268
IDR 24 after vaccination	Liver parasite load	<0.0001	-0.7902	0.6245
IDR 48 after vaccination	Liver parasite load	<0.0001	-0.7314	0.5350
INF-γ after vaccination	Liver parasite load	0.0005	-0.5501	0.3026
TNF-α after vaccination	Liver parasite load	<0.0001	-0.7204	0.5190
TNF-α/IL-10 after vaccination	Liver parasite load	0.0042	-0.4661	0.2173
Liver relative weight	Liver parasite load	0.0133	0.4088	0.1671
Spleen relative weight	Liver parasite load	0.0142	0.4053	0.1643

Additionally, the increases of IDR responses after vaccination (**Supplementary Figures S6a–d**) and the secretion of IFN-γ and TNF-α after infection (**Supplementary Figures S6e–h**) were negatively correlated with the relative weights of spleens and livers (**Table 1**). Furthermore, the IDR responses and the levels of IFN-γ, TNF-α and the TNF-α/IL-10 cytokine ratios after vaccination (**Supplementary Figures S7a–g**) were also negatively correlated to the liver LDU values, with all of them representing strong correlates of protection (**Table 2**). In contrast, as expected for disease markers, the liver parasite load was positively correlated with the relative spleen and liver weights (**Supplementary Figures S7f, g**) (**Table 2**).

## 4 Discussion

We aimed to develop a vaccine based on a highly conserved antigen in all species of the *Leishmania* genus. Such a vaccine would be advantageous because it could achieve cross-protection and prevent all types of leishmaniasis. With this purpose, we previously demonstrated that the F1F3 chimera promoted in mice an optimized vaccine efficacy against the heterologous infections by *L.* (*L*.) *amazonensis* (10), agent of diffuse cutaneous leishmaniasis, and *L.* (*V*.) *braziliensis* (22), agent of cutaneous and mucocutaneous leishmaniasis of America. In these two previous studies, as described here in the mice model for VL, the F3 and the F1F3 were the most potent vaccines (10, 22). However, while as expected after the homologous challenge with *L.* (*L*.) *infantum chagasi* the increases of anti-NH36 antibody absorbances promoted by the F3 and the F1F3 vaccines were higher and more pronounced for the IgG class followed by the IgG1, IgG2a, IgG2b, and IgG3 subclasses, after the heterologous challenge by *L.* (*V*.) *braziliensis* the increases were more substantial for the IgA class (99% and 122%, respectively) followed by the IgG2a (20% and 59%, respectively) and IgG3 subclasses (46% and 59%, respectively) (22). The predominance of an anti-NH36 IgA response after the challenge with *L.* (*V*.) *braziliensis* is a significant achievement, considering that *L.* (*V*.) *braziliensis* infection targets the skin and mucosal tissues and that IgA antibodies are the most relevant in the protection of mucosal tissues against microbial infections (26). In agreement, after the heterologous challenge with *L.* (*L*.) *amazonensis*, the antibody increases were lower than after infection with *L.* (*L*.) *infantum chagasi*, but also mainly promoted by the F1F3 chimera, as detected in IgG2a (36%), IgM (15%), and IgA (5%) increases of antibody absorbances (10). Protection against leishmaniasis is mainly related to the cellular T cell rather than to the B cell and antibody responses (27), and vaccine antibodies in VL are only a surrogate of protection (28). However, our antibody results illustrate the generation of a cross-reactivity and indicate that a vaccine against VL based on the F1F3 chimera could also generate antibodies against agents of cutaneous leishmaniasis and behave in endemic areas as a bivalent transmission-blocking vaccine (TBV) (29, 30) against both VL and CL. Transmission-blocking vaccines are essential for blocking the spread of insect-borne infectious diseases in endemic areas. This concept is derived from the development of anti-malaria vaccines (31). The antibodies generated by the vaccine in a human or animal host, once ingested by the vector, might impede the development of the pathogen inside their guts, avoiding the infection of more individuals. In this way, the vaccine might stop the epidemic cycle.

The IgG2a subclass is generally associated with a Th1(IFN-γ) response (32). In agreement, in our study, IgG2a, IFN-γ, and TNF-α ratios correlate negatively with liver parasite load and with spleen and liver relative weight, suggesting that these variables are positively associated and are markers of the Th1 response. However, no significant correlation between them was detected. On the other hand, the IgG1 subclass is commonly associated with a Th2 (IL-10) response (32, 33). However, in our study, and as observed for IgG2a, IgG1 correlated negatively with spleen and liver relative weight and with parasite load, suggesting that it is also more associated with a Th1 than with a Th2 response. In agreement, the combined enhancement of IgG2a and IgG1 is commonly described as an effect of QS21 saponin adjuvants, which are considered stimulants of mixed Th1/Th2 responses (32, 34), that still promote strong immune-protection against VL. Saponins can promote either pro-inflammatory Th1/Th2 or only anti-inflammatory Th2 immunity depending on the structure–activity relationships of their moieties (34). Therefore, high levels of both IgG1 and IgG2 are expected when using saponin (25, 35). While total IgG indicates adjuvanticity, it cannot distinguish Th1 from Th2 immunity (34). In our study, however, the IgG2a/IgG1 ratios after vaccination and after infection strongly suggest a Th1 response.

Furthermore, the response might be related not only to the adjuvant but to the antigen epitopes since, as we described before (10, 22) and in the present study, the F1F3 chimera and the F1, but not the F3 component, promote mixed IFN-γ, TNF-α, and IL-10 responses.

The intradermal response to the leishmanial antigen is a strong correlate of protection against VL (15, 28, 36–38). Only the vaccines containing F3 domains promoted an IDR response against *Leishmania* antigen. As an indication of important optimization of the vaccine efficacy and cross-protection, the F1F3 chimera was superior to the F3 and to the F1 + F3 mixture after challenge with *L.* (*L*.) *infantum chagasi*, as it was described after infections with *L.* (*L*.) *amazonensis* (10) and *L.* (*V*.) *braziliensis* (22).

In line with the increase in IDR response, the chimeras also promoted the highest IFN-γ and TNF-α inflammatory cytokine secretions before and after challenge with *L.* (*L*.) *infantum chagasi*. The highest IFN-γ secretion was observed in mice vaccinated with the chimeras, and the highest TNF-α levels were in mice immunized with chimeras or the F3 vaccine. Conversely, the F1F3 chimeras and F1 elicited a significant secretion of IL-10, whereas the F3 vaccine did not induce such a response. These differences determined a Th1 response after challenge only in mice vaccinated with F3, with enhanced IFN-γ/IL-10 and TNF-α/IL-10 ratios. In contrast, low ratios indicative of a regulatory profile were found in mice immunized with the F1F3 chimera. In agreement with that, after infection by *L.* (*V*.) *braziliensis*, the chimeras also generated stronger IDR responses and secretions of IFN-γ, TNF-α, and IL-10, suggesting a regulatory profile. At the same time, the F3 domain showed high ratios of cytokines IFN-γ/IL-10 and TNF-α/IL-10 (22), suggesting a Th1 type response. After infection by *L.* (*L*.) *amazonensis*, the IFN-γ and TNF-α secretion was also the highest in mice vaccinated with the chimeras (10). On the other hand, after infection by *L.* (*L*.) *amazonensis* and as observed in our investigation, the mixture (F1 + F3) was not as effective as the chimera in the generation of a cellular immune response (10). These results suggest that the expression of the F1 and F3 domains in tandem in the recombinant chimera F1F3 optimizes the generation of the immune response and promotes strong protection against the parasite (10).

The Th1 response in VL is associated with the production of IL-2, IL-12, IFN-γ, TNF-α, nitric oxide (NO), and reactive oxygen species (ROS). It contributes to protection from infection (11, 39), while the Th2 immune response, characterized by the production of IL-4, IL-10, TGF-β, IL-6, and others, is associated with disease progression and with parasite growth (40). Patients with active VL are immunosuppressed and do not respond to the *Leishmanin* skin test. The severe VL in humans is associated with increased levels of IL-10. On the other hand, the increased secretion of IFN-γ and TNF-α by CD4^+^ and CD8^+^ T cells is related to the cure of patients with VL (41). Furthermore, secretion of IFN-γ is restored after successful treatment (42, 43).

In contrast, however, the role of TNF-α in VL is ambivalent. It has been reported to be associated with the pathology (44–49) and its cure or protection (44, 50, 51). In hamsters vaccinated with a Th1 chimeric protein, a decrease in splenic parasite load was associated with a Th1 response against *L.* (*L*.) *donovani* infection, with high IFN-*γ* and TNF-*α* and low IL-10 secretion (51). Additionally, in mice vaccinated with the NH36 domains, increased DTH^+^ responses and ratios of TNFα/IL-10 CD4^+^ producing T cells were strong correlates of protection that induced a significant decrease in parasite burden (15). Furthermore, as a marker protection against VL, the F2 domain of NH36 induced the highest levels of IFN-γ, IL-1β, and TNF-α in DTH^+^ asymptomatic and cured subjects from Brazil (11). In addition, NH36 and the F1 domain promoted the IFN-γ and TNF-α secretion of PBMC from cured VL patients from Spain (52).

Furthermore, the chimeras also induced the highest IL-10 secretion in our study. The existence of a balance of the immune response of VL that controls active disease has been suggested (53). Prominent levels of IL-10 not counterbalanced by high levels of IFN-γ may explain the occurrence of more severe states of VL. Without considering the severity of the disease, IL-10 was present in patient sera, and low levels of IFN-γ were associated with VL severity in children (53). IL-10 can be considered an immunoregulatory cytokine, as regulatory T cells (Tregs) mediate the suppression of innate and acquired immunity cells through the secretion of IL-10, making it play an essential role in the development of VL (53). Th2, or anti-inflammatory cytokine IL-10, tends to impair the inflammatory response by producing intramonocytic IL-10 and TGF-β (54). An association between IL-10 production susceptibility of the host and progression to severe VL has also been described (55, 56). Furthermore, the splenic pathology of VL has also been correlated with high levels of TNF and interleukin IL-10. While TNF mediates the destruction of marginal zone macrophages and gp38(+) stromal cells, IL-10 determines diminished DC migration to T-cells and their priming (57).

T regulatory epitopes for mice (10, 22) and for humans (58) were described in the F3 domain and for mice in the F1 domain (10). However, two HLA-Class II epitopes that induce a strong Th1 response in mice were also described in the sequence of F3 (10, 22). This differential epitope composition might explain the predominance of a Th1-driven response generated by the F3 vaccine, which is effective against infections by *L.* (*L*.) *infantum chagasi* (24, 59), *L.* (*L*.) *amazonensis* (10, 16), and *L.* (*L*.) *braziliensis* (22). In contrast, the contribution of T regulatory epitopes of the F1 domain expressed in tandem with F3 explains the mixed regulatory response generated by the F1F3 chimera vaccine against the visceral infection by *L.* (*L*.) *infantum chagasi* in the present investigation and against the cutaneous infections by *L.* (*V*.) *braziliensis* (22) and *L.* (*L*.) *amazonensis* (10). Epitopes of NH36 that stimulate both the secretion of inflammatory and regulatory cytokines by PBMC of human patients cured from VL or asymptomatic were recently described and used in the composition of multiepitope vaccines against VL (58).

Finally, regarding the clinical outputs, the F1F3 chimera vaccine promoted the strongest gain in corporal weight, reduction of liver and spleen relative weights, and the most pronounced reduction of parasite load in livers, indicating that it induced protection against the homologous infection by *L.* (*L*.) *infantum chagasi*. As the chimeras also promoted the most significant reduction in the size of the mice paws’ lesion and ear lesions caused, respectively, by the heterologous infections with *L.* (*L*.) *amazonensis* (10) and *L.* (*V*.) *braziliensis* (22), it is possible to conclude that the F1F3 chimera determines cross-protection against VL and CL in the mice model. The F3 vaccine was as effective as the chimeras in the reduction of spleen relative weight and *L.* (*L*.) *infantum chagasi* parasite load and stronger than the F1 + F3 mixture against infection by *L.* (*L*.) *amazonensis* (10). In a previous work, we also demonstrated that the expression of the F1 and F3 fractions in tandem in the F1F3 chimera, rather than as a simple mixture, enhanced the antigen presentation to the CD8^+^ and CD4^+^ T cells, optimizing in this way the immune response generated against infection by *L.* (*L*.) *amazonensis* (10). In the case of future clinical studies of efficacy, the expression of both domains in tandem in a chimera would facilitate the production of the vaccine antigen in a pilot or scaling-up industrial scale. In contrast, the F1 vaccine was not effective in increasing corporal weight or reducing the spleen and liver relative weight and promoted a 38% lower reduction in parasite load than the chimeras in mice infected with *L.* (*L*.) *infantum chagasi*, while it did not induce any reduction in lesion sizes due to *L.* (*L*.) *amazonensis* (10) or *L.* (*V*.) *braziliensis* (22). The strong and significant correlations between the immunological variables and the clinical and parasitological outcomes in mice challenged with *L.* (*L*.) *infantum chagasi* support our conclusions.

Therefore, the recombinant F1F3 chimera remains the best choice. In addition to improving vaccine efficacy and prophylactic protection against *L.* (*L*.) *infantum chagasi*, which causes VL, it also optimizes vaccine efficacy and prophylactic protection against CL caused by *L.* (*L*.) *amazonensis* and *L.* (*V*.) *braziliensis*. Thus, it allows the design of a bivalent vaccine to be used to prevent both types of leishmaniasis: cutaneous and visceral.

## Data Availability

The original contributions presented in the study are included in the article/**Supplementary Material**, further inquiries can be directed to the corresponding author/s.

## Supplementary material

Supplementary Figure 1 SDS-PAGE analysis of the purified recombinant proteins. From left to right: low molecular weight markers (Pharmacia) (pm: 97, 66, 45, 30, 20.1, and 14.4 kDa), NH36 (32 µg), F1 (40 µg), F3 (34 µg), F1-F3lab (14.2 µg) cloned with non-optimized codons (black arrow), and F1-F3GS (28 µg) cloned with optimized codons by Genscript (red arrow), all stained with Coomassie Brilliant Blue R-250 (BioRad).

Supplementary Figure 2 The antibody absorbance values increase after infection. The mice were immunized with F3 or F1F3 chimeras at 100 or 200 ug doses, all formulated in 100 µg saponin. Increased absorbance values of anti-NH36 IgA, IgM, IgG, IgG2a, IgG2b, and IgG3 antibodies were expressed as percentages **(a)** and as means of fold increases **(b)**.

Supplementary Figure 3 Microscopic images of liver smears. Representative image of live smears of saline-treated controls **(a)** and mice vaccinated with F1F3 and saponin **(b)** after euthanasia, stained with Giemsa dye, and using a ×100 immersion objective. Images were acquired using an Axio Scope A1 optical microscope equipped with the Axiovision 4.9.1. imaging software (Carl Zeiss, Germany).

Supplementary Figure 4 Correlations between antibody levels after infection and clinical outcomes. The mice were vaccinated with the F1, F3, F1+F3 mixture, or F1F3 chimeras at 100- or 200-µg doses, all formulated in 100 µg saponin. Dots represent the individual data results of correlation between spleen/body relative weight percent and IgM **(a)**, IgG **(b)**, IgG1**(c)**, IgG2a **(d)**, IgG2b **(e)**, and IgG3 **(f)** antibody absorbance values after infection and between liver/body relative weight percent and IgM **(g)**, IgG **(h)**, IgG1**(i)**, IgG2a **(j)**, IgG2b **(k)**, and IgG3 **(l)** antibody absorbance values after infection. Correlation analyses were performed using the bivariate two-tailed Pearson test in the GraphPad 6 Prism software.

Supplementary Figure 5 Correlations between antibody levels after vaccination and clinical outcomes. The mice were vaccinated with the F1, F3, F1+F3 mixture, or F1F3 chimeras at 100- or 200-µg doses formulated in 100 µg saponin. Dots represent the individual data results of the correlation between liver parasite load in LDU values and IgA **(a)**, IgM **(b)**, IgG **(c)**, IgG1 **(d)**, IgG2a **(e)**, IgG2b **(f)**, and IgG3 **(g)** antibody absorbance values after vaccination. Correlation analyses were performed using the bivariate two-tailed Pearson test in GraphPad 6 Prism.

Supplementary Figure 6 Correlations between intradermal response to leishmanial antigen after vaccination or cytokines after infection and clinical outputs. The mice were vaccinated with the F1, F3, F1+F3 mixture, or F1F3 chimeras at 100- or 200-µg doses formulated in 100 µg saponin. Dots represent the individual data results of correlation between intradermal response to leishmanial antigen after vaccination (in mm) and spleen **(a, b)** and liver/body relative weight percent after infection **(c, d)** and of IFN-γ **(e, f)** and TNF-α concentrations (pg/mL) secreted after infection **(g, h)**, and spleen and liver/body relative weight percent, respectively, after infection. Correlation analyses were performed using the bivariate two-tailed Pearson test in GraphPad 6 Prism software.

Supplementary Figure 7 Correlations between the liver parasite load and the intradermal response to leishmanial antigen, cytokines after vaccination, and the spleen and liver relative weights. The mice were vaccinated with the F1, F3, F1+F3 mixture, or F1F3 chimeras at 100- or 200-µg doses, all formulated in 100 µg saponin. Dots represent the individual data results of correlations between the liver parasite load (LDU values) and the intradermal response to leishmanial antigen after vaccination (in mm) **(a, b)**, the IFN-γ **(c)** and TNF-α **(d)** concentrations (pg/mL), the TNF-α/IL-10 ratios **(e)** secreted after vaccination **(g, h)**, and the liver/body **(f)** and spleen/relative weight percent **(g)** after infection. Correlation analyses were performed using the bivariate two-tailed Pearson test in GraphPad 6 Prism.

## References

[1] Leishmaniasis. Available online at: https://www.who.int/news-room/fact-sheets/detail/leishmaniasis (Accessed February 25, 2025).

[2] Le Rutte EA Coffeng LE Malvolti S Kaye PM de Vlas SJ. The potential impact of human visceral leishmaniasis vaccines on population incidence. PLoS Negl Trop Dis. (2020) 14:e0008468. doi: 10.1371/journal.pntd.0008468 32614857 PMC7363103

[3] van Griensven J Diro E. Visceral leishmaniasis: recent advances in diagnostics and treatment regimens. Infect Dis Clin North Am. (2019) 33:79–99. doi: 10.1016/j.idc.2018.10.005 30712769

[4] Palatnik-de-Sousa CB Day MJ. One Health: the global challenge of epidemic and endemic leishmaniasis. Parasit Vectors. (2011) 4:197. doi: 10.1186/1756-3305-4-197 21985335 PMC3214158

[5] Carvalho LS das Graças Braga M da Silva Costa DA Simões TC Lula MD Silveira MR. Lethality among individuals infected with visceral leishmaniasis in Brazil: a retrospective study (2007-2018). Parasitol Res. (2022) 121:725–36. doi: 10.1007/s00436-022-07429-3 35013872

[6] Burza S Croft SL Boelaert M. Leishmaniasis. Lancet Lond Engl. (2018) 392:951–70. doi: 10.1016/S0140-6736(18)31204-2 30126638

[7] Palatnik-de-Sousa CB. Nucleoside hydrolase NH 36: A vital enzyme for the leishmania genus in the development of T-cell epitope cross-protective vaccines. Front Immunol. (2019) 10:813. doi: 10.3389/fimmu.2019.00813 31040850 PMC6477039

[8] Lukes J Mauricio IL Schönian G Dujardin JC Soteriadou K Dedet JP. Evolutionary and geographical history of the Leishmania donovani complex with a revision of current taxonomy. Proc Natl Acad Sci U S A. (2007) 104:9375–80. doi: 10.1073/pnas.0703678104 PMC1890502 17517634

[9] Mauricio IL Yeo M Baghaei M Doto D Pratlong F Zemanova E. Towards multilocus sequence typing of the Leishmania donovani complex: resolving genotypes and haplotypes for five polymorphic metabolic enzymes (ASAT, GPI, NH1, NH2, PGD). Int J Parasitol. (2006) 36:757–69. doi: 10.1016/j.ijpara.2006.03.006 16725143

[10] Alves-Silva MV Nico D Morrot A Palatnik M Palatnik-de-Sousa CB. A Chimera Containing CD4+ and CD8+ T-Cell Epitopes of the Leishmania donovani Nucleoside Hydrolase (NH36) Optimizes Cross-Protection against Leishmania amazonesis Infection. Front Immunol. (2017) 8:100. doi: 10.3389/fimmu.2017.00100 28280494 PMC5322207

[11] Barbosa Santos ML Nico D de Oliveira FA Barreto AS Palatnik-de-Sousa I Carrillo E. Leishmania donovani nucleoside hydrolase (NH36) domains induce T-cell cytokine responses in human visceral leishmaniasis. Front Immunol. (2017) 8:227. doi: 10.3389/fimmu.2017.00227 28321221 PMC5338038

[12] Coler RN Duthie MS Hofmeyer KA Guderian J Jayashankar L Vergara J. From mouse to man: safety, immunogenicity and efficacy of a candidate leishmaniasis vaccine LEISH-F3+GLA-SE. Clin Transl Immunol. (2015) 4:e35. doi: 10.1038/cti.2015.6 PMC4488838 26175894

[13] Cui L Rajasekariah GR Martin SK. A nonspecific nucleoside hydrolase from Leishmania donovani: implications for purine salvage by the parasite. Gene. (2001) 280:153–62. doi: 10.1016/s0378-1119(01)00768-5 11738828

[14] BLAST: basic local alignment search tool. Available online at: https://blast.ncbi.nlm.nih.gov/Blast.cgi (Accessed February 9, 2025).

[15] Nico D Claser C Borja-Cabrera GP Travassos LR Palatnik M Soares I da S. Adaptive immunity against Leishmania nucleoside hydrolase maps its c-terminal domain as the target of the CD4+ T cell-driven protective response. PLoS Negl Trop Dis. (2010) 4:e866. doi: 10.1371/journal.pntd.0000866 21085470 PMC2976684

[16] Nico D Gomes DC Alves-Silva MV Freitas EO Morrot A Bahia D. Cross-Protective Immunity to Leishmania amazonensis is Mediated by CD4+ and CD8+ Epitopes of Leishmania donovani Nucleoside Hydrolase Terminal Domains. Front Immunol. (2014) 5:189. doi: 10.3389/fimmu.2014.00189 24822054 PMC4013483

[17] Kao DJ Hodges RS. Advantages of a synthetic peptide immunogen over a protein immunogen in the development of an anti-pilus vaccine for Pseudomonas aeruginosa. Chem Biol Drug Des. (2009) 74:33–42. doi: 10.1111/j.1747-0285.2009.00825.x 19519742 PMC2756486

[18] Palatnik-de-Sousa CB Soares I da S Rosa DS. Editorial: epitope discovery and synthetic vaccine design. Front Immunol. (2018) 9:826. doi: 10.3389/fimmu.2018.00826 29720983 PMC5915546

[19] Lopes KF Freire ML Murta SMF Oliveira E. Efficacy of vaccines based on chimeric or multiepitope antigens for protection against visceral leishmaniasis: A systematic review. PLoS Negl Trop Dis. (2024) 18:e0012757. doi: 10.1371/journal.pntd.0012757 39739955 PMC11753665

[20] Parody N Soto M Requena JM Alonso C. Adjuvant guided polarization of the immune humoral response against a protective multicomponent antigenic protein (Q) from Leishmania infantum. A CpG + Q mix protects Balb/c mice from infection. Parasite Immunol. (2004) 26:283–93. doi: 10.1111/j.0141-9838.2004.00711.x 15541032

[21] Fernández Cotrina J Iniesta V Monroy I Baz V Hugnet C Marañon F. A large-scale field randomized trial demonstrates safety and efficacy of the vaccine LetiFend^®^ against canine leishmaniosis. Vaccine. (2018) 36:1972–82. doi: 10.1016/j.vaccine.2018.02.111 29525281

[22] Alves-Silva MV Nico D de Luca PM Palatnik de-Sousa CB. The F1F3 Recombinant Chimera of Leishmania donovani-Nucleoside Hydrolase (NH36) and Its Epitopes Induce Cross-Protection Against Leishmania (V.) Braziliensis Infection in Mice. Front Immunol. (2019) 10:724. doi: 10.3389/fimmu.2019.00724 31024556 PMC6465647

[23] Bradley DJ Kirkley J. Regulation of Leishmania populations within the host. I. @ the variable course of Leishmania donovani infections in mice. Clin Exp Immunol. (1977) 30:119–29. PMC1541173 606433

[24] Nico D Martins Almeida F Maria Motta J Soares Dos Santos Cardoso F Freire-de-Lima CG Freire-de-Lima L. NH36 and F3 antigen-primed dendritic cells show preserved migrating capabilities and CCR7 expression and F3 is effective in immunotherapy of visceral leishmaniasis. Front Immunol. (2018) 9:967. doi: 10.3389/fimmu.2018.00967 29867949 PMC5949526

[25] Santos WR de Lima VMF de Souza EP Bernardo RR Palatnik M Palatnik de Sousa CB. Saponins, IL12 and BCG adjuvant in the FML-vaccine formulation against murine visceral leishmaniasis. Vaccine. (2002) 21:30–43. doi: 10.1016/s0264-410x(02)00444-9 12443660

[26] de Sousa-Pereira P Woof JM. IgA: structure, function, and developability. Antibodies (Basel). (2019) 8:57. doi: 10.3390/antib8040057 31817406 PMC6963396

[27] Tiwari R Kumar A Singh VK Rajneesh Chauhan SB Sundar S. The development and maintenance of immunity against visceral leishmaniasis. Front Immunol. (2024) 15:1486407. doi: 10.3389/fimmu.2024.1486407 39781380 PMC11707418

[28] Plotkin SA. Correlates of protection induced by vaccination. Clin Vaccine Immunol. (2010) 17:1055–65. doi: 10.1128/CVI.00131-10 PMC2897268 20463105

[29] Saraiva EM de Figueiredo Barbosa A Santos FN Borja-Cabrera GP Nico D Souza LOP. The FML-vaccine (Leishmune) against canine visceral leishmaniasis: a transmission blocking vaccine. Vaccine. (2006) 24:2423–31. doi: 10.1016/j.vaccine.2005.11.061 16386824

[30] Palatnik-de-Sousa CB Barbosa A de F Oliveira SM Nico D Bernardo RR Santos WR. FML vaccine against canine visceral leishmaniasis: from second-generation to synthetic vaccine. Expert Rev Vaccines. (2008) 7:833–51. doi: 10.1586/14760584.7.6.833 18665780

[31] Duffy PE. Transmission-blocking vaccines: harnessing herd immunity for malaria elimination. Expert Rev Vaccines. (2021) 20:185–98. doi: 10.1080/14760584.2021.1878028 PMC11127254 33478283

[32] Lacaille-Dubois MA. Updated insights into the mechanism of action and clinical profile of the immunoadjuvant QS-21: A review. Phytomedicine. (2019) 60:152905. doi: 10.1016/j.phymed.2019.152905 31182297 PMC7127804

[33] Guy B. The perfect mix: recent progress in adjuvant research. Nat Rev Microbiol. (2007) 5:505–17. doi: 10.1038/nrmicro1681 17558426

[34] Marciani DJ. Elucidating the mechanisms of action of saponin-derived adjuvants. Trends Pharmacol Sci. (2018) 39:573–85. doi: 10.1016/j.tips.2018.03.005 29655658

[35] Palatnik de Sousa CB Santos WR Casas CP Paraguai de Souza E Tinoco LW da Silva BP. Protective vaccination against murine visceral leishmaniasis using aldehyde-containing Quillaja saponaria sapogenins. Vaccine. (2004) 22:2470–9. doi: 10.1016/j.vaccine.2004.01.072 15193411

[36] Stober CB Jeronimo SMB Pontes NN Miller EN Blackwell JM. Cytokine Responses to Novel Antigens in a Peri-Urban Population in Brazil Exposed to Leishmania infantum chagasi. Am J Trop Med Hyg. (2012) 87:663–70. doi: 10.4269/ajtmh.2012.12-0180 PMC3516316 22826477

[37] Abánades DR Arruda LV Arruda ES Pinto JRAS Palma MS Aquino D. Immunodominant antigens of Leishmania chagasi associated with protection against human visceral leishmaniasis. PLoS Negl Trop Dis. (2012) 6:e1687. doi: 10.1371/journal.pntd.0001687 22724032 PMC3378602

[38] Nico D Feijó DF Maran N Morrot A Scharfstein J Palatnik M. Resistance to visceral leishmaniasis is severely compromised in mice deficient of bradykinin B2-receptors. Parasit Vectors. (2012) 5:261. doi: 10.1186/1756-3305-5-261 23151408 PMC3514163

[39] Yadav S Prakash J Singh OP Gedda MR Chauhan SB Sundar S. IFN-γ+ CD4+ T cell-driven prophylactic potential of recombinant LDBPK_252400 hypothetical protein of Leishmania donovani against visceral leishmaniasis. Cell Immunol. (2021) 361:104272. doi: 10.1016/j.cellimm.2020.104272 33445051 PMC7890570

[40] Samant M Sahu U Pandey SC Khare P. Role of cytokines in experimental and human visceral leishmaniasis. Front Cell Infect Microbiol. (2021) 11:624009. doi: 10.3389/fcimb.2021.624009 33680991 PMC7930837

[41] Rodrigues LS Barreto AS Bomfim LGS Gomes MC Ferreira NLC da Cruz GS. Multifunctional, TNF-α and IFN-γ-secreting CD4 and CD8 T cells and CD8High T cells are associated with the cure of human visceral leishmaniasis. Front Immunol. (2021) 12:773983. doi: 10.3389/fimmu.2021.773983 34777391 PMC8581227

[42] Achour A Derouiche A Driss MR Tebourbi O. Organochlorine pesticides (OCPs) and polychlorinated biphenyls (PCBs) in adipose tissue of women from Grand Tunis and their association with demographic factors and dietary habits. Chemosphere. (2023) 338:139600. doi: 10.1016/j.chemosphere.2023.139600 37480958

[43] de Franca MNF Rodrigues LS Barreto AS da Cruz GS Aragão-Santos JC da Silva AM. CD4+ Th1 and Th17 responses and multifunctional CD8 T lymphocytes associated with cure or disease worsening in human visceral leishmaniasis. Front Immunol. (2024) 15:1277557. doi: 10.3389/fimmu.2024.1277557 38410517 PMC10895669

[44] Zwingenberger K Harms G Pedrosa C Pessoa MC Sandkamp B Scheibenbogen C. Generation of cytokines in human visceral leishmaniasis: dissociation of endogenous TNF-α and IL-1β Production. Immunobiology. (1991) 183:125–32. doi: 10.1016/s0171-2985(11)80192-0 1937561

[45] Araújo-Santos T Andrade BB Gil-Santana L Luz NF Dos Santos PL de Oliveira FA. Anti-parasite therapy drives changes in human visceral leishmaniasis-associated inflammatory balance. Sci Rep. (2017) 7:4334. doi: 10.1038/s41598-017-04595-8 28659627 PMC5489532

[46] Peruhype-Magalhães V Martins-Filho OA Prata A Silva LDA Rabello A Teixeira-Carvalho A. Mixed inflammatory/regulatory cytokine profile marked by simultaneous raise of interferon-gamma and interleukin-10 and low frequency of tumour necrosis factor-alpha(+) monocytes are hallmarks of active human visceral Leishmaniasis due to Leishmania chagasi infection. Clin Exp Immunol. (2006) 146:124–32. doi: 10.1111/j.1365-2249.2006.03171.x PMC1809731 16968407

[47] Dos Santos PL de Oliveira FA Santos MLB Cunha LCS Lino MTB de Oliveira MFS. The severity of visceral leishmaniasis correlates with elevated levels of serum IL-6, IL-27 and sCD14. PLoS Negl Trop Dis. (2016) 10:e0004375. doi: 10.1371/journal.pntd.0004375 26814478 PMC4729473

[48] de Medeiros IM Castelo A Salomão R. Presence of circulating levels of interferon-gamma, interleukin-10 and tumor necrosis factor-alpha in patients with visceral leishmaniasis. Rev Inst Med Trop Sao Paulo. (1998) 40:31–4. doi: 10.1590/s0036-46651998000100007 9713135

[49] Barral-Netto M Badaró R Barral A Almeida RP Santos SB Badaró F. Tumor necrosis factor (cachectin) in human visceral leishmaniasis. J Infect Dis. (1991) 163:853–7. doi: 10.1093/infdis/163.4.853 1901333

[50] Dayakar A Chandrasekaran S Kuchipudi SV Kalangi SK. Cytokines: key determinants of resistance or disease progression in visceral leishmaniasis: opportunities for novel diagnostics and immunotherapy. Front Immunol. (2019) 10:670. doi: 10.3389/fimmu.2019.00670 31024534 PMC6459942

[51] Ratnapriya S Keerti Yadav NK Dube A Sahasrabuddhe AA. A Chimera of Th1 Stimulatory Proteins of Leishmania donovani Offers Moderate Immunotherapeutic Efficacy with a Th1-Inclined Immune Response against Visceral Leishmaniasis. BioMed Res Int. (2021) 2021:8845826. doi: 10.1155/2021/8845826 34095312 PMC8164546

[52] Carrillo E Fernandez L Ibarra-Meneses AV Santos MLB Nico D de Luca PM. F1 Domain of the Leishmania (Leishmania) donovani Nucleoside Hydrolase Promotes a Th1 Response in Leishmania (Leishmania) infantum Cured Patients and in Asymptomatic Individuals Living in an Endemic Area of Leishmaniasis. Front Immunol. (2017) 8:750. doi: 10.3389/fimmu.2017.00750 28747911 PMC5506215

[53] Gama MEA Gomes CM Silveira FT Laurenti MD Gonçalves Eda G da Silva AR. Severe visceral leishmaniasis in children: the relationship between cytokine patterns and clinical features. Rev Soc Bras Med Trop. (2013) 46:741–5. doi: 10.1590/0037-8682-0203-2013 24474016

[54] Roy S Mukhopadhyay D Mukherjee S Moulik S Chatterji S Brahme N. An IL-10 dominant polarization of monocytes is a feature of Indian Visceral Leishmaniasis. Parasite Immunol. (2018) 40:e12535. doi: 10.1111/pim.12535 29745990

[55] Nylén S Maurya R Eidsmo L Manandhar KD Sundar S Sacks D. Splenic accumulation of IL-10 mRNA in T cells distinct from CD4+CD25+ (Foxp3) regulatory T cells in human visceral leishmaniasis. J Exp Med. (2007) 204:805–17. doi: 10.1084/jem.20061141 PMC2118563 17389235

[56] Gautam S Kumar R Maurya R Nylén S Ansari N Rai M. IL-10 neutralization promotes parasite clearance in splenic aspirate cells from patients with visceral leishmaniasis. J Infect Dis. (2011) 204:1134–7. doi: 10.1093/infdis/jir461 PMC3164427 21881130

[57] Stanley AC Engwerda CR. Balancing immunity and pathology in visceral leishmaniasis. Immunol Cell Biol. (2007) 85:138–47. doi: 10.1038/sj.icb7100011 17146466

[58] Barreto AS Franca MNF dos Reis TLDS Silva JABM Santos P Oliveira FA. Design and development of highly conserved, HLA-promiscuous T cell multiepitope vaccines against human visceral Leishmaniasis. Front Immunol. (2025) 16:1540537. doi: 10.3389/fimmu.2025.1540537 40230841 PMC11994619

[59] Palatnik-de-Sousa CB. Nucleoside hydrolase NH 36: A vital enzyme for the leishmania genus in the development of T-cell epitope cross-protective vaccines. Front Immunol. (2019) 10:813. doi: 10.3389/fimmu.2019.00813 31040850 PMC6477039

