# Supplementary material for: The F1F3 recombinant chimera induced higher vaccine efficacy than its independent F1 and F3 components against Leishmania (L.) infantum chagasi mice infection
Source: Front Immunol. 2025 Jul 1;16:1598755. doi: 10.3389/fimmu.2025.1598755 (PMC12260408; doi:10.3389/fimmu.2025.1598755)

Figure S1. SDS-PAGE analysis of the recombinant antigens

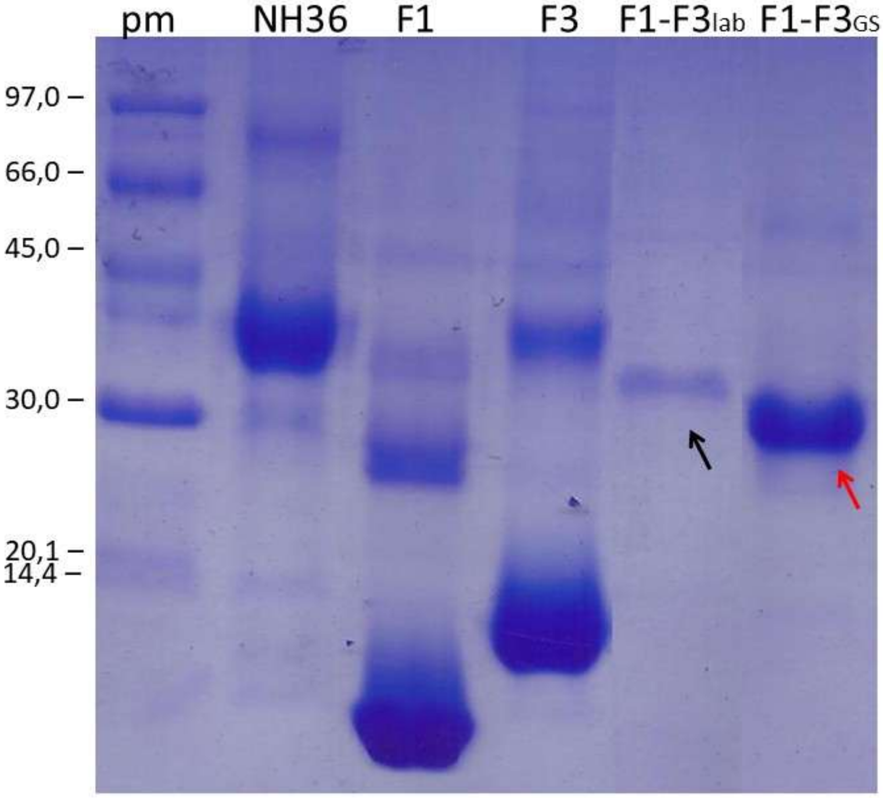

Figure S2. Antibody absorbance values increase after infection.

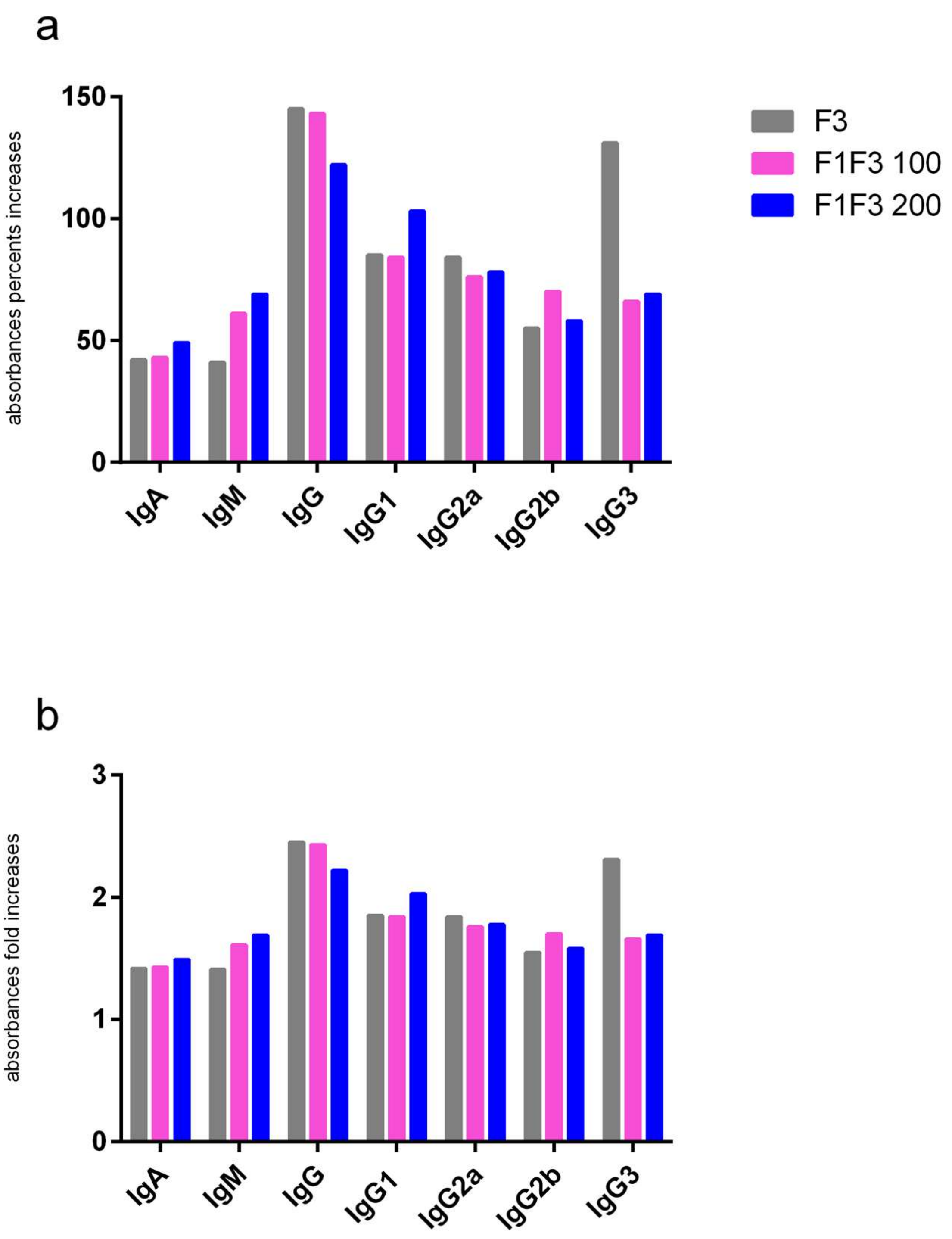

Figure S3. Representative images of microscopical observation of liver-smears of control (a) and F1F3 vaccinated mice (b).

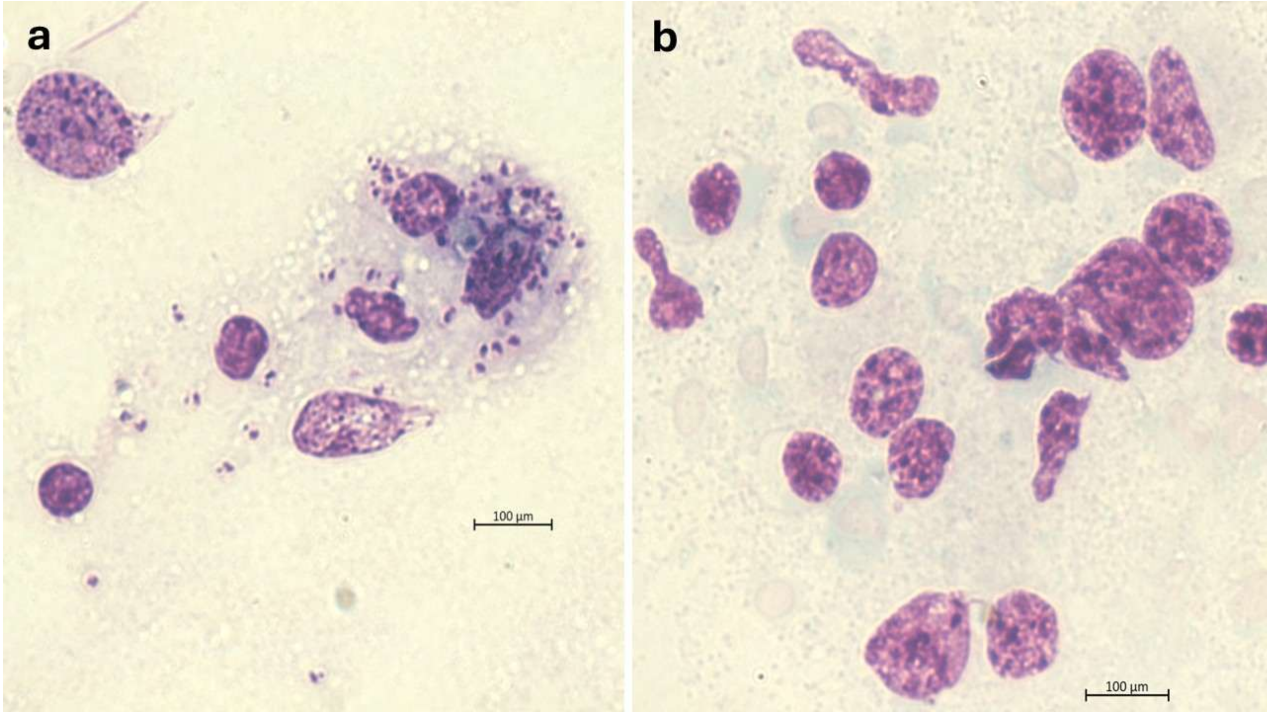

Figure S4. Correlations between antibody levels after infection and clinical outcomes.

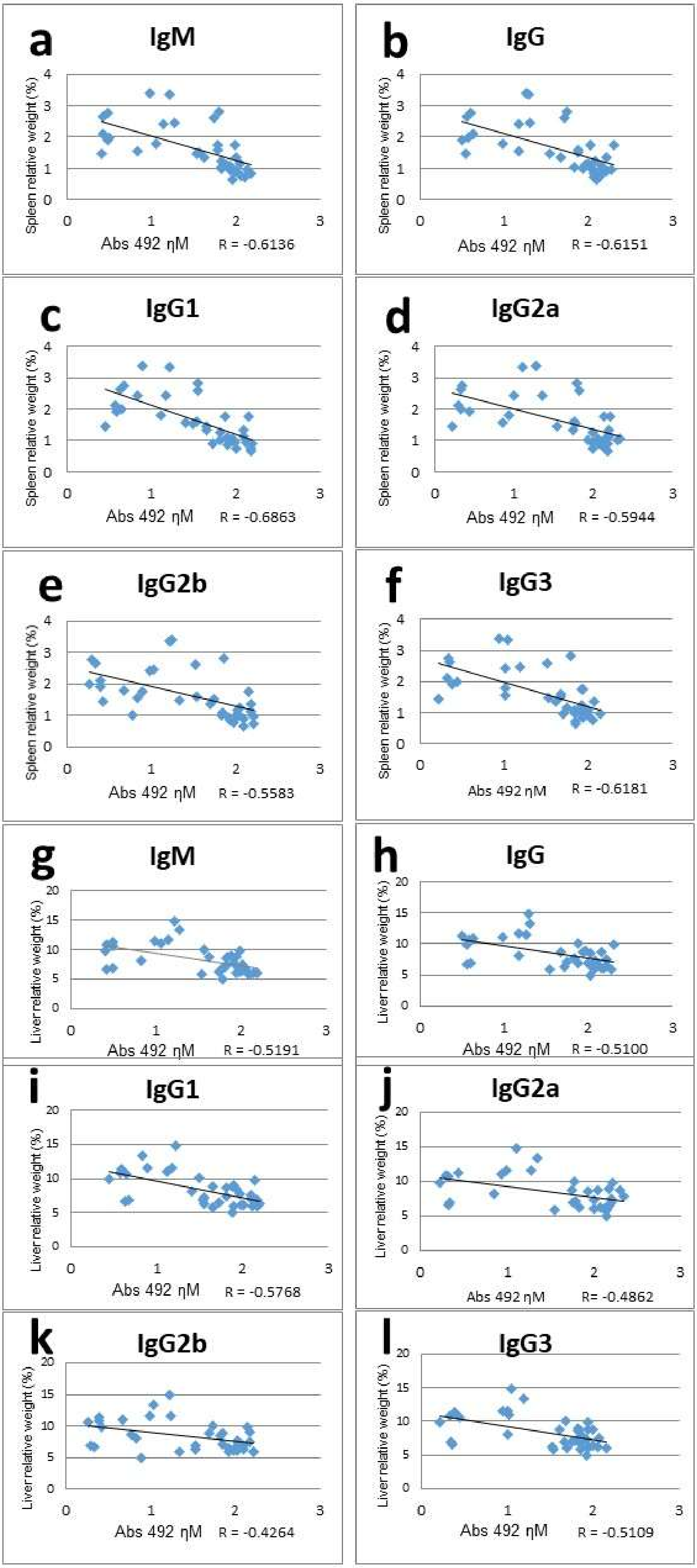

Figure S5. Correlations between antibody levels after vaccination and clinical outcomes.

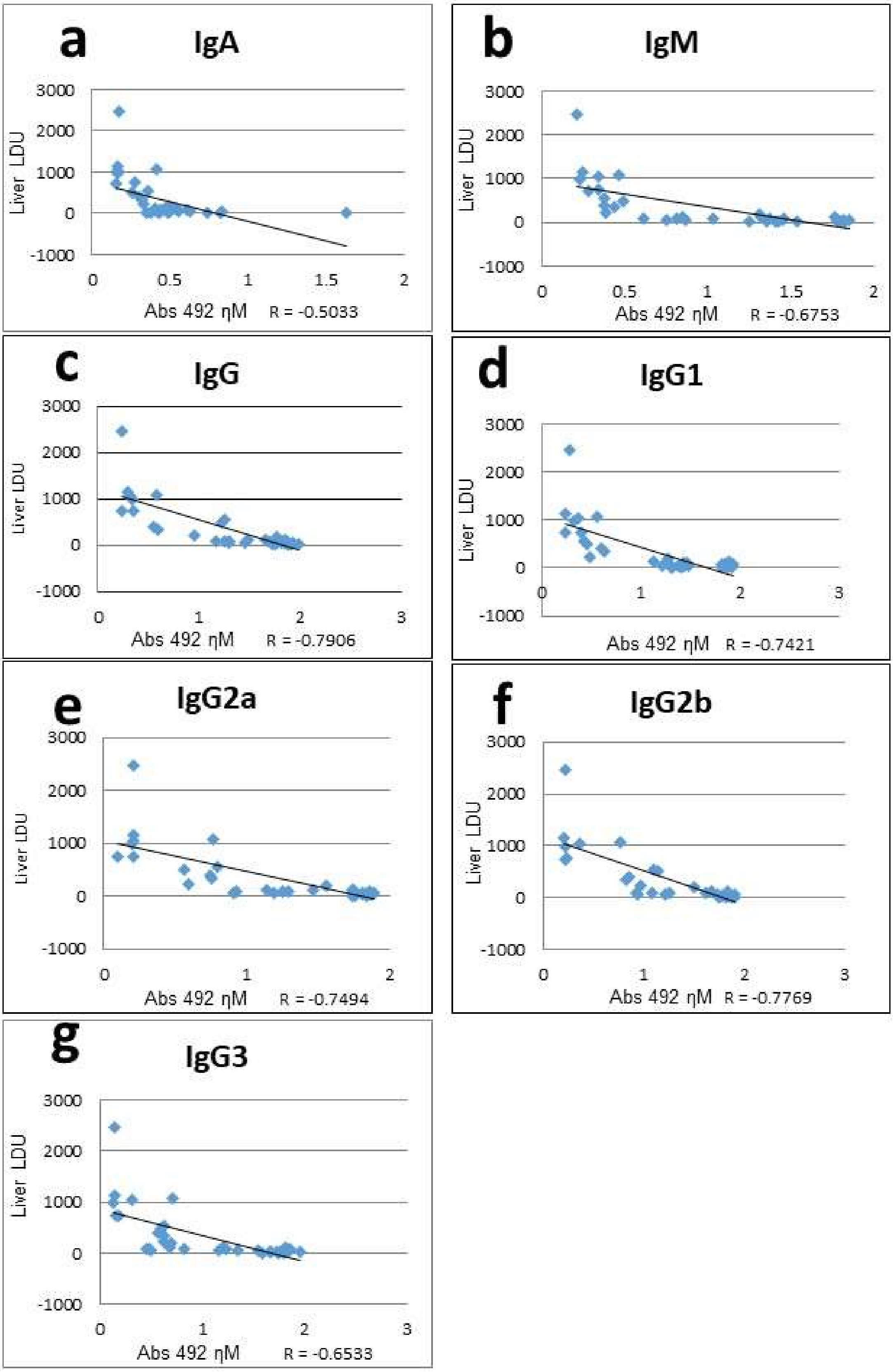

Figure S6. Correlations between intradermal response to leishmanial antigen after vaccination or cytokines after infection and clinical outcomes.

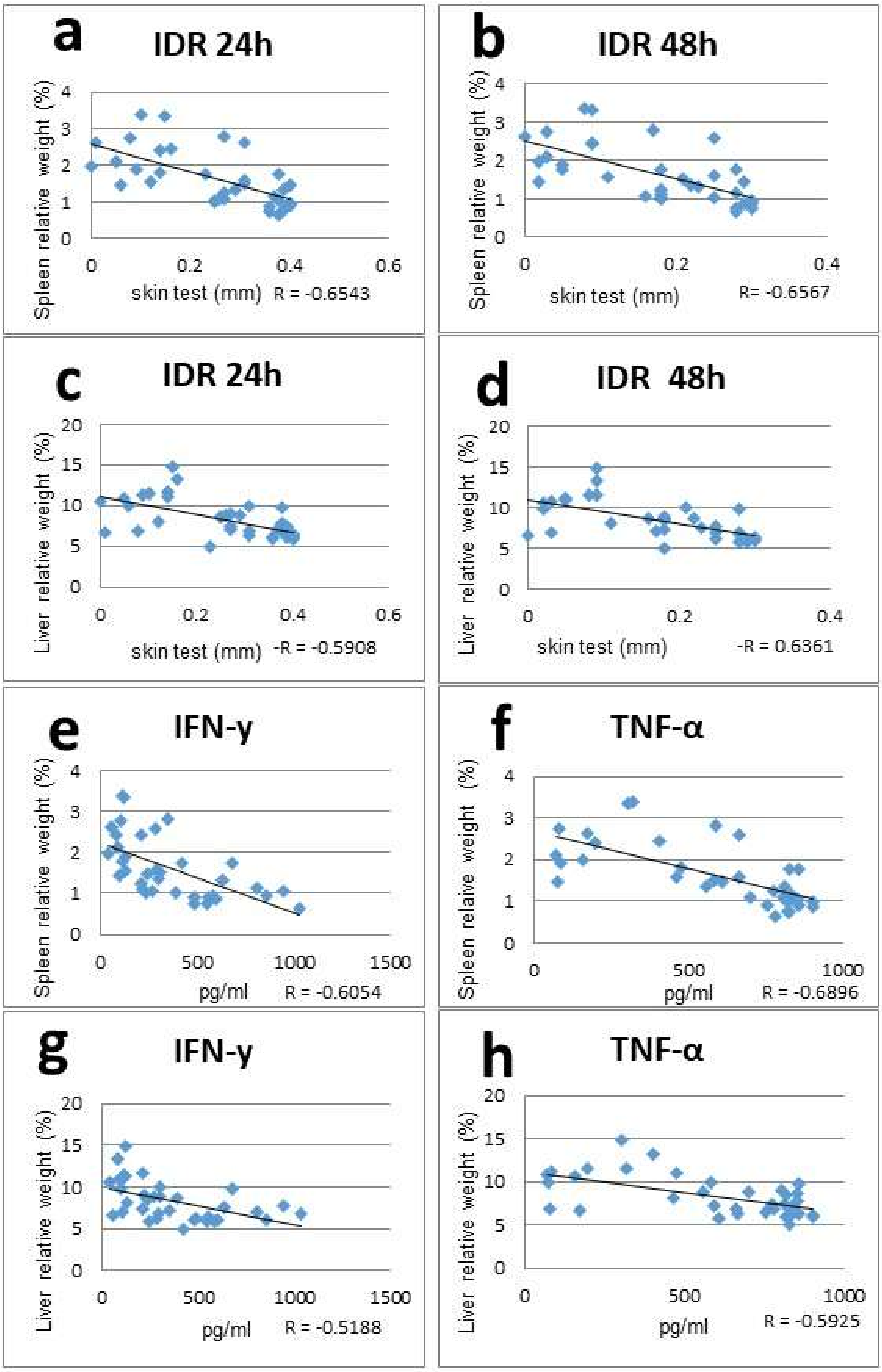

Figure S7. Correlations between the liver parasite load and the intradermal response to leishmanial antigen, cytokines after vaccination and the spleen and liver relative weights.

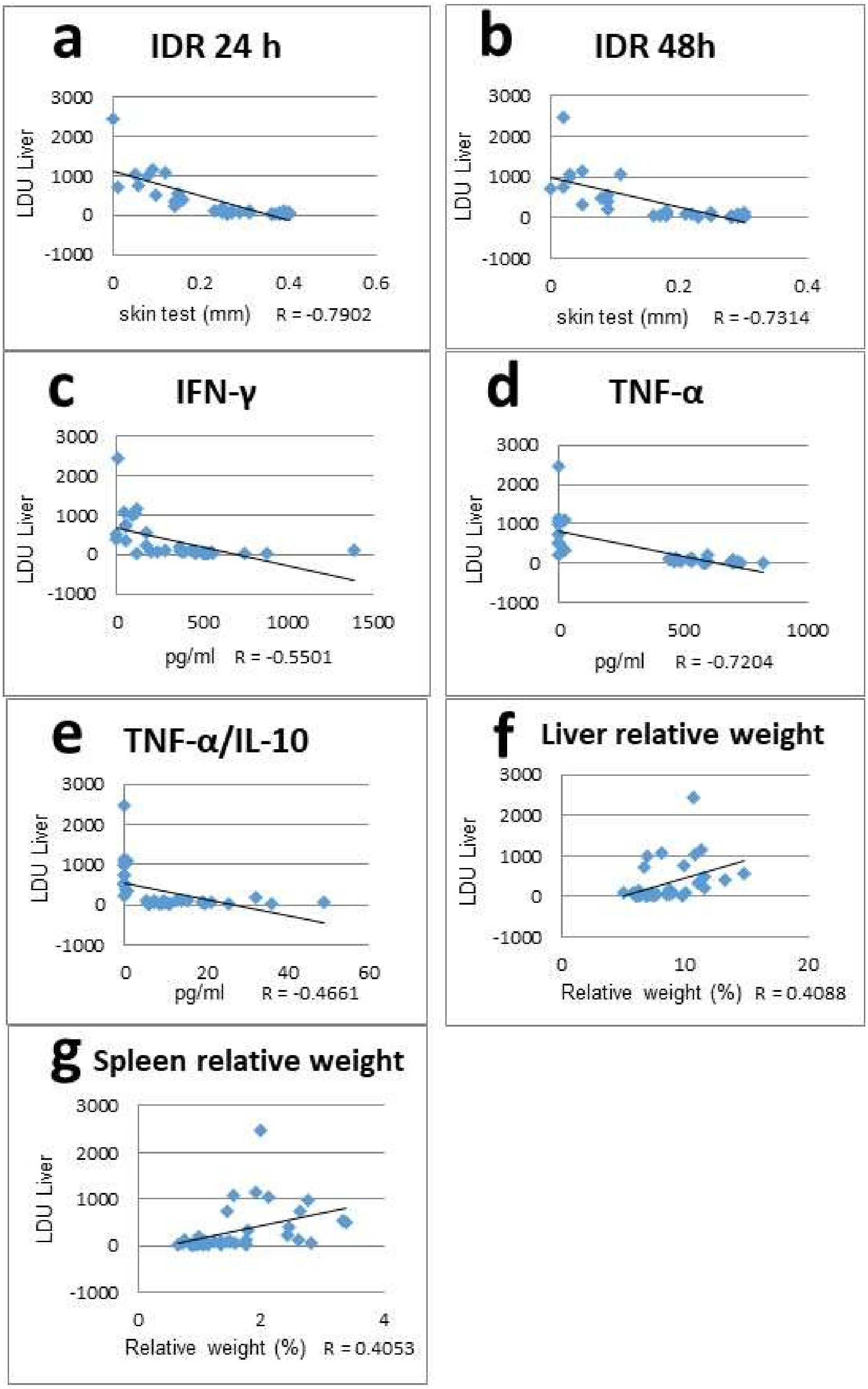

Supplement: Supplementary Figure 1 — SDS-PAGE analysis of the purified recombinant proteins. From left to right: low molecular weight markers (Pharmacia) (pm: 97, 66, 45, 30, 20.1, and 14.4 kDa), NH36 (32 µg), F1 (40 µg), F3 (34 µg), F1-F3lab (14.2 µg) cloned with non-optimized codons (black arrow), and F1-F3GS (28 µg) cloned with optimized codons by Genscript (red arrow), all stained with Coomassie Brilliant Blue R-250 (BioRad). [file DataSheet1.pdf]
